# Water-flooding characteristics of lithologic reservoir in Ordos basin

**DOI:** 10.1038/s41598-021-82035-4

**Published:** 2021-01-28

**Authors:** Jie He, Xiaodong Liu, Xinyu Zhu, Tao Jiang, Hui He, Lin Zhou, Qinghai Liu, Yushuang Zhu, Linyu Liu

**Affiliations:** 1 State Key Laboratory of Continental Dynamics, Department of Geology, Northwest University, Xi’an, 710069 China; 2 No. 7 Oil Production Plant, PetroChina Changqing Oilfield Company, Xifeng, 745000 Gansu China; 3 No. 1 Oil Production Plant, PetroChina Daqing Oilfield Company, Daqing, 163001 Heilongjiang China

**Keywords:** Energy science and technology, Engineering

## Abstract

Due to the poor situation of water-flooding mechanism research on Chang 4 + 5 reservoir of Ordos basin, the authors quantitatively studied the influence factors of water-flooding characteristics by sedimentology, casting thin sections, constant-speed mercury injection, scanning electron microscope as well as production records. The size and distribution of pore-throat were also found closely related with the water-flooding seepage law. The results show that the microscopic seepage paths of Chang 4 + 5 reservoir include uniform displacement, finger displacement and peak displacement, and their correspondent oil displacement efficiency reduces in turn under the same conditions. Reservoir heterogeneity, reservoir properties, distribution of pore structure and wettability play a decisive role in water-flooding efficiency. Generally, When the intra-layer range is greater than 4.65, the breakthrough coefficient is greater than 3.54, the coefficient of variation is greater than 0.7, the distribution frequency of inter-layer is greater than 0.5 per meter, and the distribution density is greater than 0.435%, the range between layers is greater than 6.86, the breakthrough coefficient is greater than 2.58, the coefficient of variation is greater than 0.51, and the thickness of inter-layer is greater than 7.54 m. the increasing trend of oil displacement efficiency will be obviously weakened.

## Introduction

At present, the Yanchang formation in Jiyuan area of Ordos Basin is a research hot spot in the research of water-flooding development of low permeability and tight sandstone reservoirs at home and abroad. The exploration and development practice of Changqing Oilfield shows that the reservoir characteristics and water-flooding law of this kind of reservoir are difficult to find, and the degree of oil and water recovery of adjacent wells is quite different^1,2^. For the study of reservoir characteristics, according to physical properties and pore structure^3,4^. ^5^concluded that the lower the permeability and the stronger the heterogeneity in the layer are, the more complex the effect of water-flooding is. On the basis of reservoir core study and logging interpretation, ^6^concluded that the effect of water-flooding is inversely proportional to the physical properties of the reservoir. By means of thin section observation and scanning electron microscope. ^7^concluded that intragranular and intergranular dissolution pores are the main types of oil and gas storage, and micro-fractures and fractures are the main migration channels. ^8^studied the factors affecting the characteristics of water flooding in low permeability reservoirs by means of constant-speed mercury injection, nuclear magnetic resonance and thin section observation. For the study of reservoir water-flooding mechanism and influencing factors, ^9^carried out constant pressure water-flooding experiments with different heterogeneity models, which showed that the greater the permeability is, the faster the water cut increased, and the higher the oil displacement efficiency is. The greater the inter-layer permeability difference is, the lower the oil displacement efficiency is. These research results highlight the characteristics and micro-seepage of reservoir, however, there are few studies on the law of water-flooding in the whole reservoir, and there are few reports on the analysis of water-flooding characteristics based on reservoir production performance data^10–12^.

At present, the water-flooding efficiency of Chang 4 + 5 reservoir in Jiyuan area of Ordos Basin is low, and the research on reservoir characteristics, water flooding characteristics and influencing factors is relatively poor. In this paper, taking Chang 4 + 5 in Jiyuan oilfield as an example, representative core samples are selected, and the microscopic characteristics of reservoir are discussed by physical properties, constant-speed mercury injection, scanning electron microscope and thin section observation, and production performance data. The dynamic and static combination method is used to analyze the relationship between reservoir characteristics and water-flooding law, and the key factors affecting oil displacement efficiency are identified to provide guidance for the development of this kind of reservoir in high water cut stage.

## Reservoir sedimentary background of Jiyuan Oilfield

**Figure 1:**
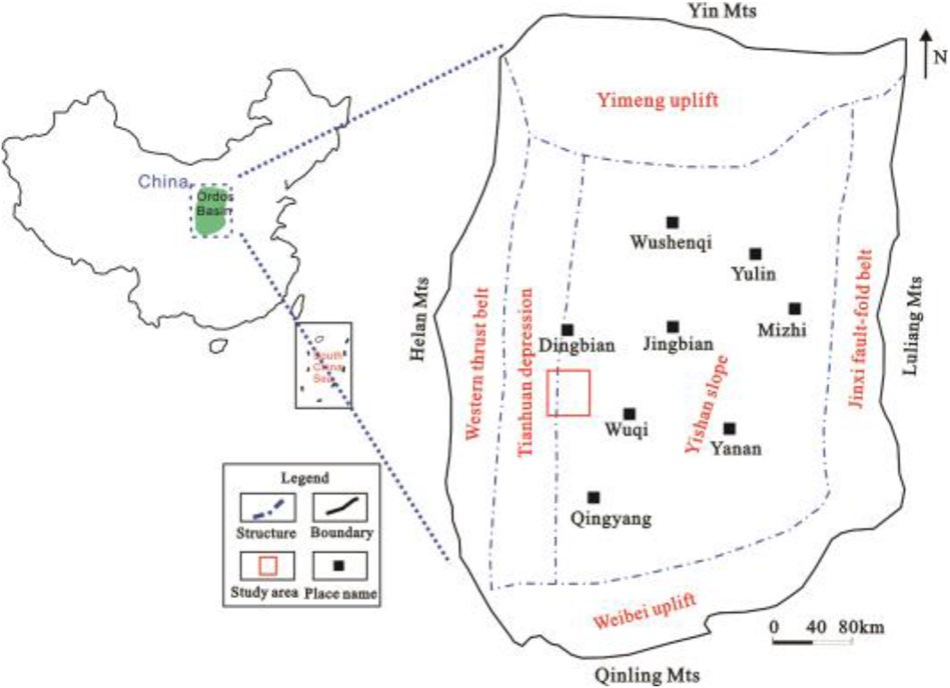
Location of study area. Created using CorelDRAW-X7 17.1.0.572 (https://www.coreldraw.com/cn/).

**Figure 2:**
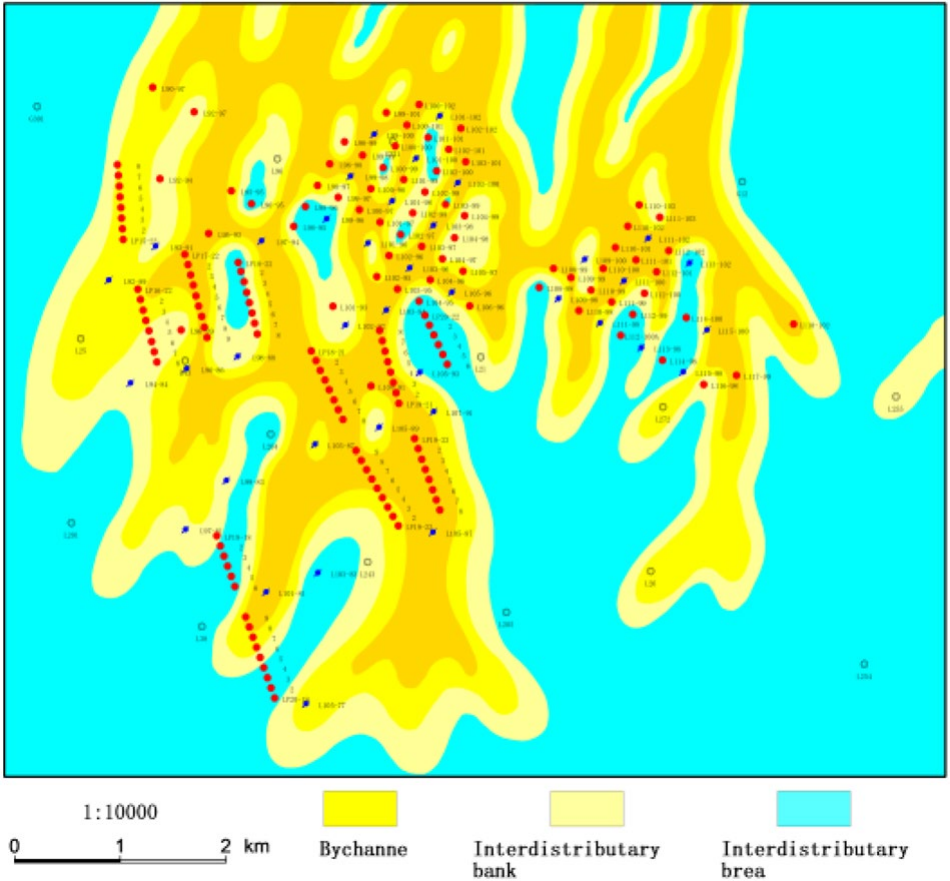
Sedimentary facies of study area. Created using GeoMap v3.6 (http://www.jurassic.com.cn/).

**Figure 3:**
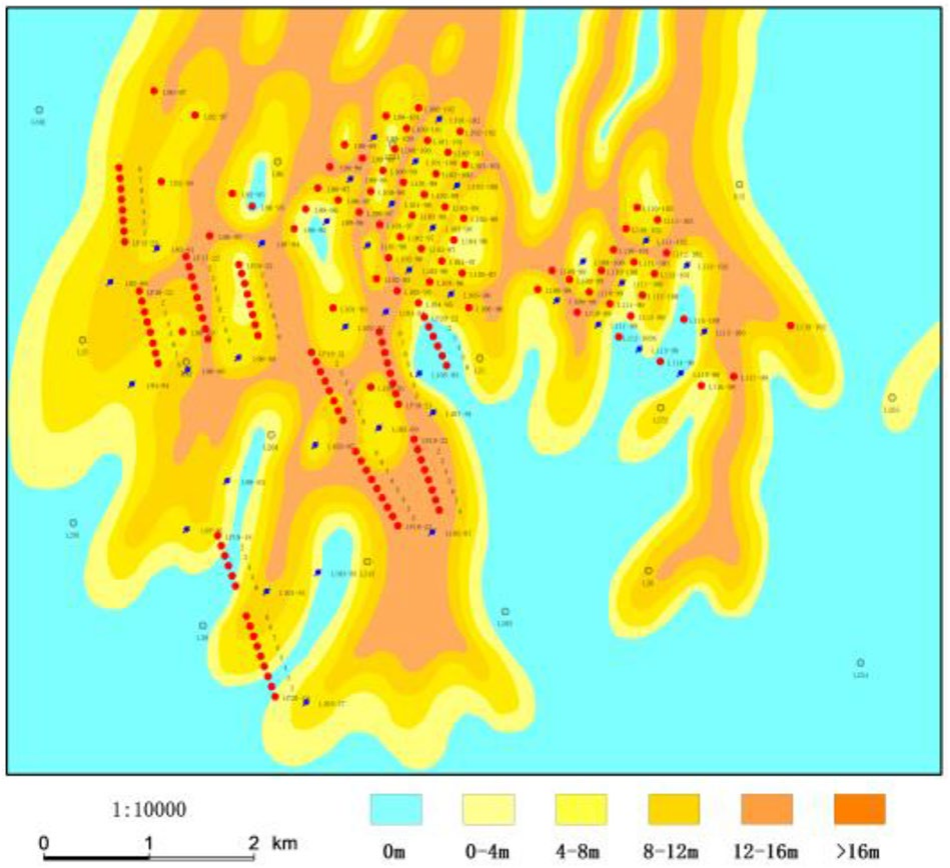
Sand body of study area. Created using GeoMap v3.6 (http://www.jurassic.com.cn/).

**Figure 4:**
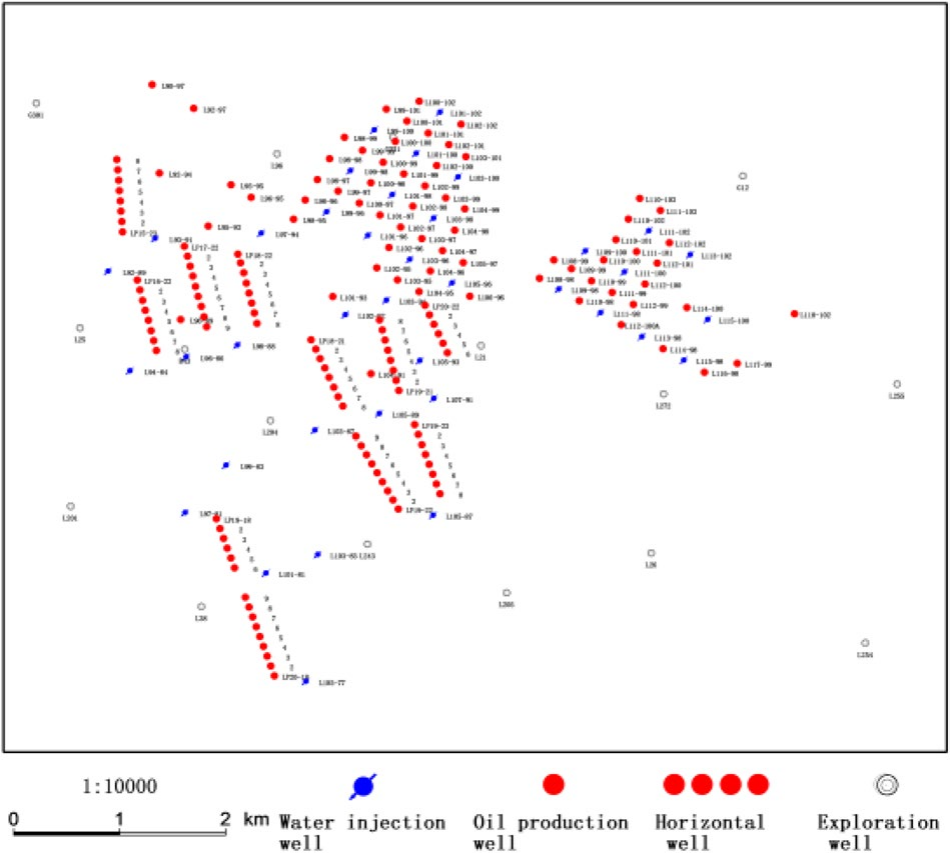
Well location map. Created using GeoMap v3.6 (http://www.jurassic.com.cn/).

Jiyuan Oilfield is located in the west of Ordos Basin, and Luo 21 area is located in the south of Jiyuan Oilfield (Fig. 1)^13,14^. The study area is the deposition of braided river delta front, which mainly consists of bychannel, interdistributary area and so on (Fig. 2). The overall connectivity of the sand body along the provenance direction is good, showing a continuous and banded distribution in the profile, and the main superimposed sand body is mainly developed; the overall connectivity of the sand body in the vertical provenance direction is slightly poor, and the main body is zonal distribution (Fig. 3) (see Supplementary Information 1).The target layer Chang 4 + 5 in the study area is the main petroliferous bed of the study area, which is composed of two subsegments: Chang 4 + 5_1_ and Chang4 + 5_2_. The target layer in the study area is divided into 8 single sand body-level research sublayers, Chang 4 + 5_1_^1^, Chang 4 + 5_1_^2^, Chang 4 + 5_13_^3^, Chang 4 + 5_1_^4^, Chang 4 + 5_2_^5^, Chang 4 + 5_2_^2^, Chang 4 + 5_2_^3^, Chang 4 + 5_2_^4^ and the thin layer is about 10 cm–17 m. The main reservoirs Chang 4 + 5 and Chang 4 + 5 are typical low permeability reservoirs. It was put into development in 2012. The total number of oil and water wells is 132 (of which 67 are oil wells) (Fig. 4). The geological reserves developed is 803.59 × 10^4^t, the daily oil production is 58.55t, the water cut is 44.59%, the recovery percent is 15.86%, the production rate is 0.25%, and the daily water injection is 309.5 m^3^. In the process of water-flooding, the injected water flows along relatively high porosity, high permeability or micro-fractures, resulting in small sweep coefficient, low oil displacement efficiency, short water-free production period. The water cut of sand layer in some production wells rises rapidly in the early stage and even serious water flooding is prominent^15^.

## Method

### Geologic setting

**Figure 5:**
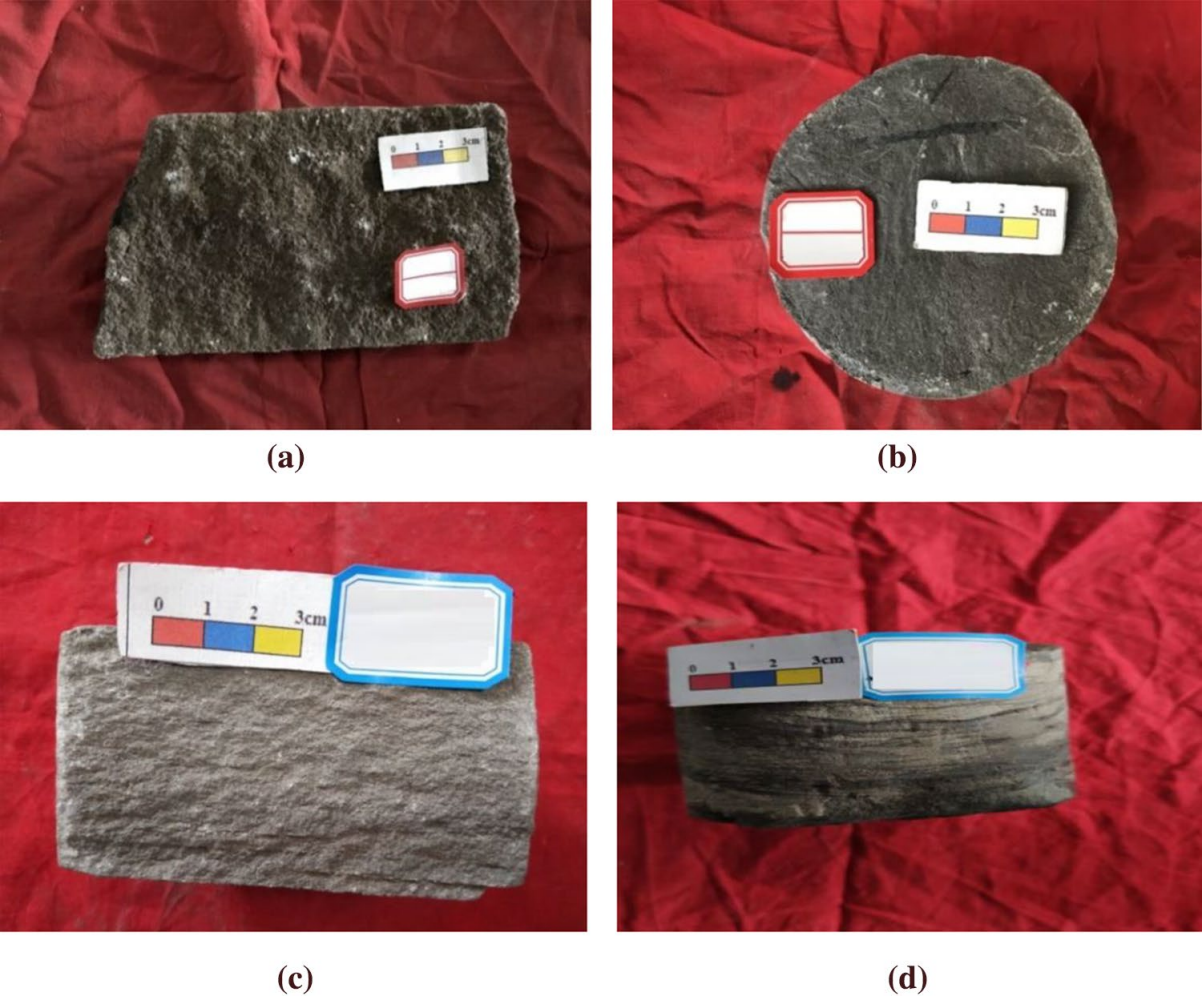
Analysis of Sedimentary characteristics of coring Wells. L255, Chang 4 + 5_1_^2^, 2258.05 m, massive bedding; (**b**) L115–100, Chang 4 + 5_2_^2^, 2166.83 m, biogenic structure; (**c**) L21, Chang 4 + 5_2_^2^, 2103.1 m, parallel bedding; (**d**) L21, Chang 4 + 5_2_^3^, 2113.43 m, deformation structure.

In the evolution process from occurrence to extinction of the late Triassic lake basin in the Ordos basin, it experienced early initial subsidence, accelerated expansion, lake basin shrinkage, and finally lake basin extinction^16,17^. After the formation of Chang 7, Chang 4 + 5 is another important period of formation of source rocks in the basin^18,19^. The sedimentary pattern shows that the shoreline of the lake moves outward and the scope of lake transgression expands^20^. The eastern and northeastern deltas, especially the northern of Ansai delta, have been largely leveled, mainly by distributary channel deposits, and the northern part has been leveled and swamped^21^. The Shigouyi fan delta in the western margin continues to exist, while the Fuxian delta and Huangling delta extend to the west of Hulu River-Taibai and to the deep lake area of Guchengchuan area, which becomes the main material source of this area. The results of coring well data show that the common sedimentary structures in the study area mainly include deformation structure, bedding plane structure, bedding structure and biogenic structure (Fig. 5)^22^.

### Petrological characteristics

**Figure 6:**
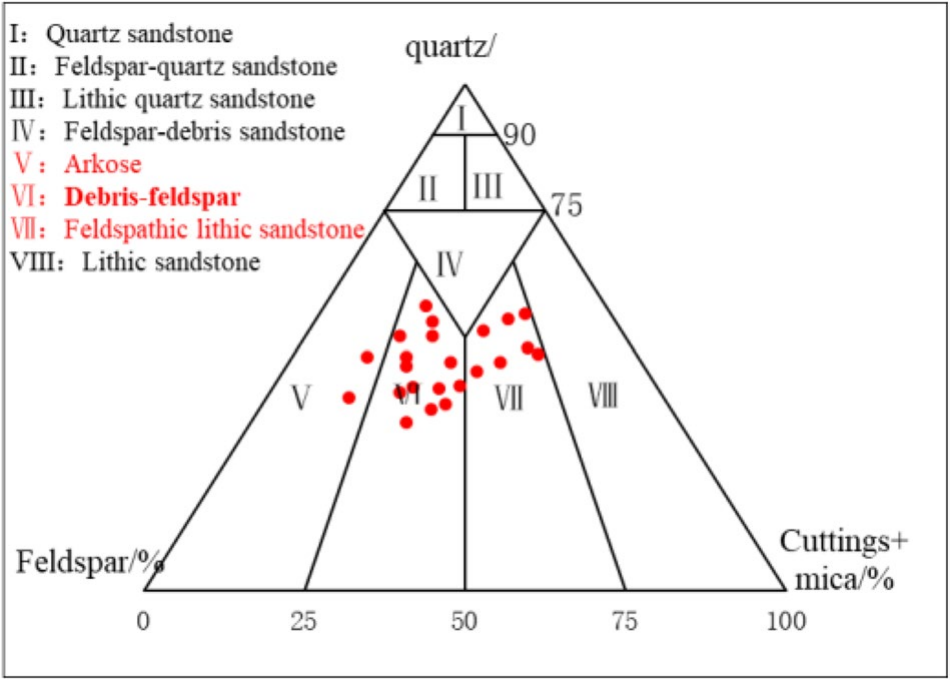
The classification of Chang 4 + 5 reservoir sandstone in Jiyuan area (see Supplementary Information 1). Created using OriginPro 2019 (https://www.originlab.com/).

**Figure 7:**
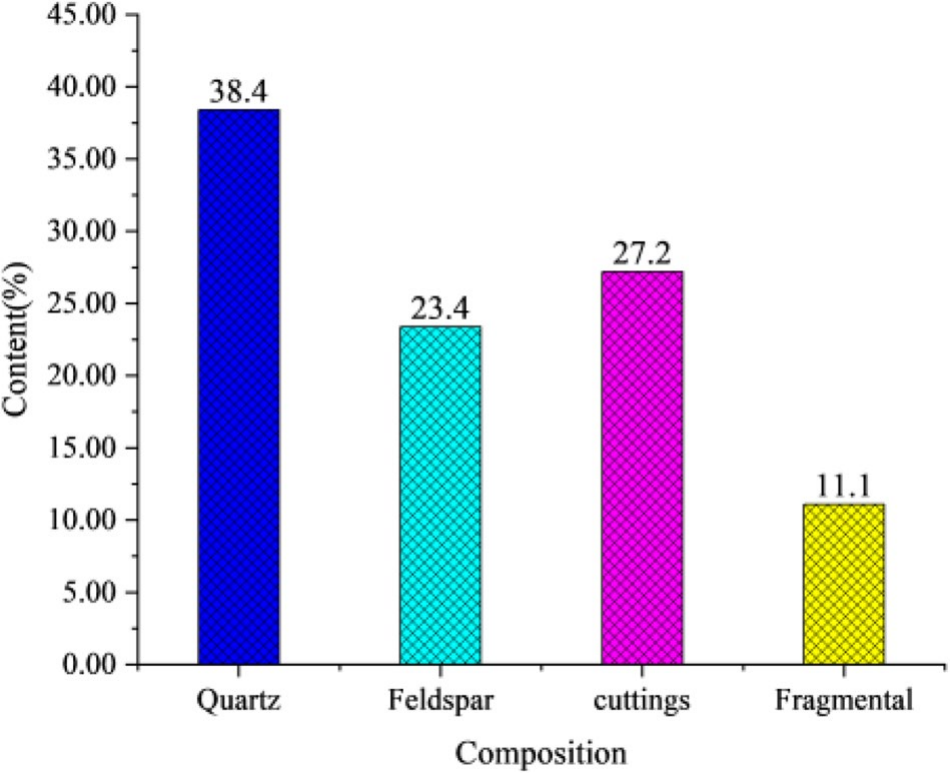
Clastic composition of reservoir sandstone in Luo 21 area.

According to the core observation in the field and the experimental analysis of the casting thin sections in the laboratory, The rock mineral composition of Chang 4 + 5 reservoir in the study area is mainly gray fine-grained debris-feldspar, containing a small amount of feldspathic lithic sandstone and feldspathic sandstone (Fig. 6)^23^. In the clastic material composition, the mass fraction of quartz is 38%, the feldspar is 23.4%, the cuttings is 27.2%, and interstitial material is 11.1% (Fig. 7). The cementation type is mainly pore-cementation, and the support type is mainly grain-supported, which reflects the strong compaction. The psephicity is sub-circular and sub-prismatic, the separability is medium-good, and the main particle size is 0.1–0.4 mm. Based on the above analysis, the compositional maturity and textural maturity of reservoir sandstone in the study area are low.

**Figure 8:**
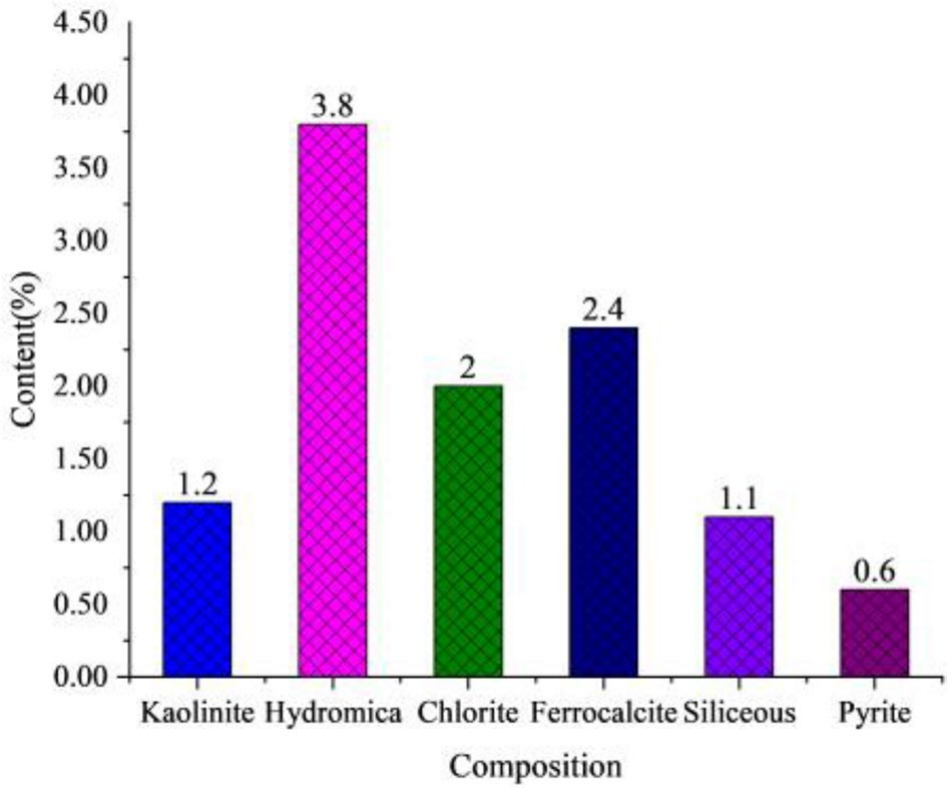
Content histogram of interstitial material in reservoir sandstone in Luo 21 area.

**Figure 9:**
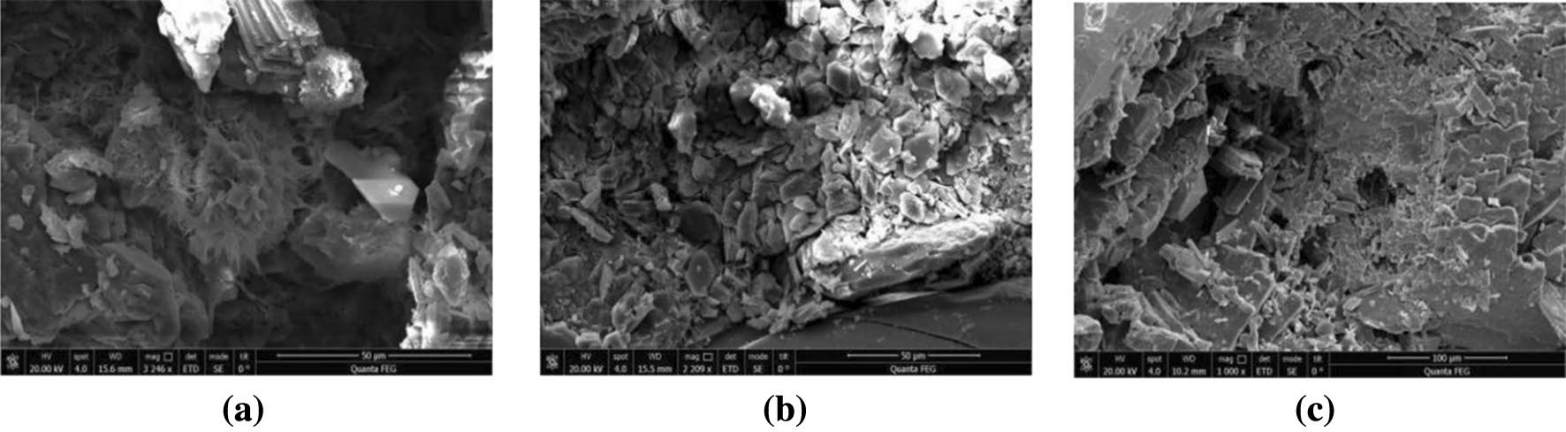
Typical pore types of Chang 4 + 5 sandstones, Jiyuan Oil Field, Ordos Basin. (**a**) Illite interstitial material (L255, 2256.75 m, Chang 4 + 5_1_^2^); (**b**) kaolinite interstitial material (L21, 2103.1 m, Chang 4 + 5_2_^2^); (**c**) quartz (L115-100, 2160.4 m, Chang 4 + 5_2_^2^).

The interstitial material consists of clay minerals (7%), carbonate cements (2.4%), siliceous cements (1.1%) and pyrite (0.6%). Clay minerals are mainly hydromica (3.8%) and chlorite (2.0%), containing a small amount of kaolinite (1.2%) (Fig. 8). The main component of carbonate cement is ferrocalcite (2.4%). Siliceous cement (1.1%) generally appears in two forms, one of which is microcrystalline quartz filling pores and the other is filled in the pore space in the form of secondary enlargement of quartz particles. A small amount of pyrite was also found in the casting thin section of the study area (Fig. 9).

### Pore types, pore structure

The microscopic pore structure of rock is an important content in the study of reservoir characteristics^24–26^. By studying the microscopic pore structure of the reservoir, the distribution and characteristics of rock pore, throat, pore-throat can be obtained, which is beneficial to the comprehensive evaluation of the reservoir^27,28^.

#### Pore types

**Figure 10:**
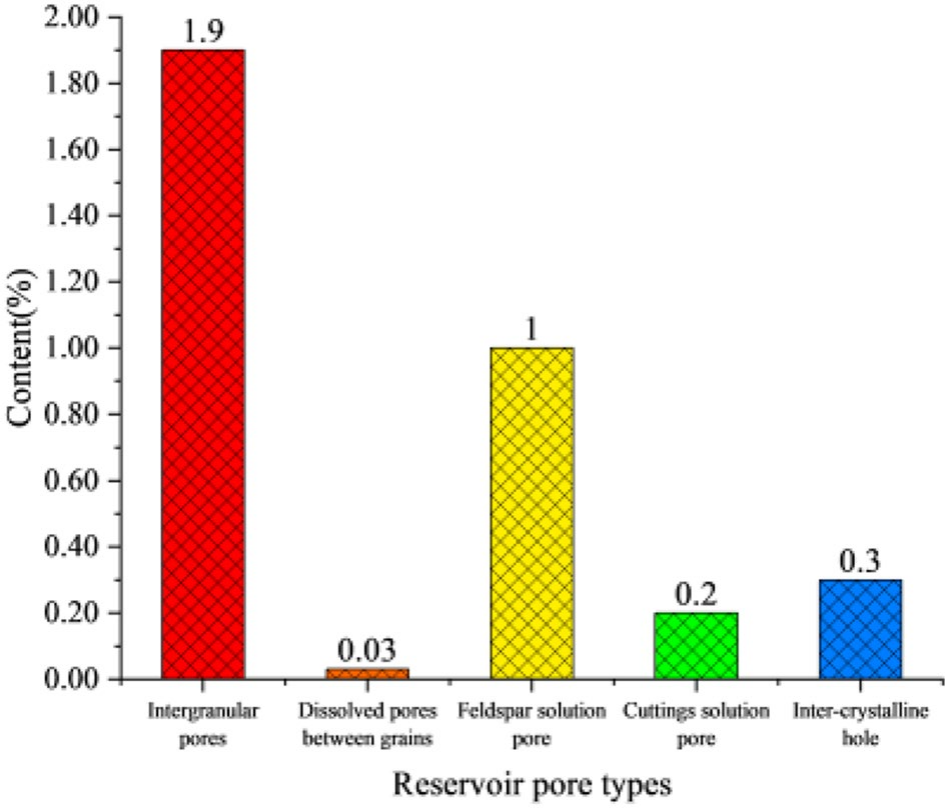
Distribution histogram of reservoir pore types in Luo 21 area.

**Figure 11:**
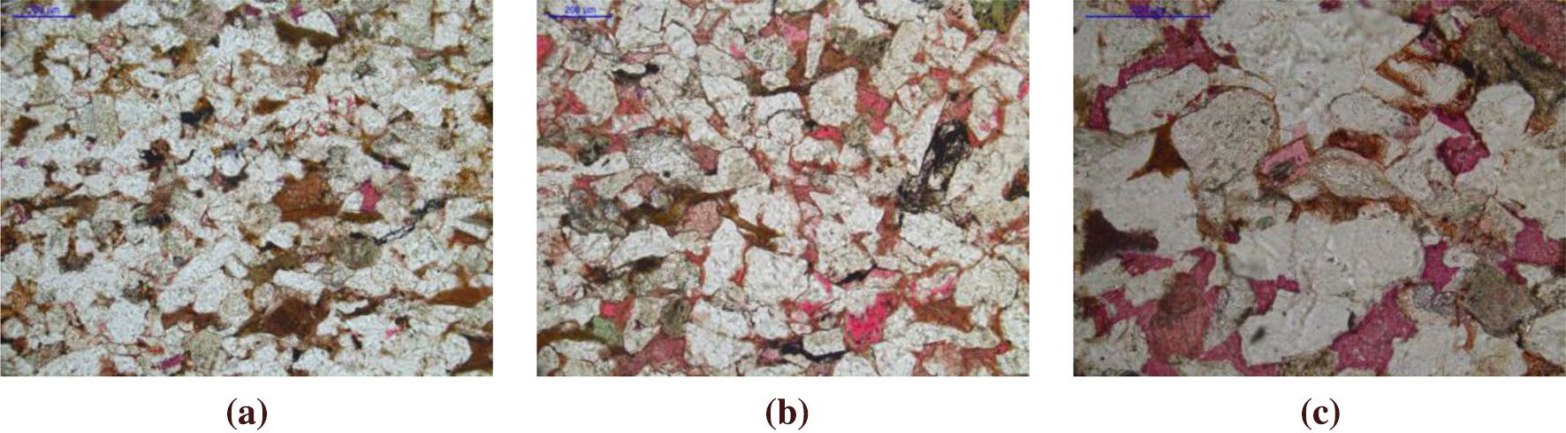
Typical cast thin sections of Chang 4 + 5 reservoir in Jiyuan oil field. (**a**) Intergranular pores (L21, 2103.1 m, Chang 4 + 5_2_^2^); (**b**) feldspar solution pores, cuttings solution pores (L255, 2256.75 m, Chang 4 + 5_1_^2^); (**c**) intergranular solution pores (L115-100, 2160.4 m, Chang 4 + 5_2_^2^).

Intergranular pores are the remaining pores after compaction and filling cementation, which are partly filled by impurities and cements, with triangular, polygonal and irregular shapes, which have the greatest influence on reservoir porosity. Through the observation of casting thin sections, and scanning electron microscopy, the pore types in the study area are mainly composed of intergranular pores (1.9%), feldspar solution pores (1.0%), cuttings solution pores (0.2%), intergranular pores (0.3%), and intergranular solution pores (0.03%) (Fig. 10). The average pore diameter is 18.75 μm, and the average area percent of pore is 3.4% (Table 1). The pore assemblage type is mainly dissolution pores-intergranular pores (Fig. 11).

**Table 1:** Statistics of pore structure characteristic parameters of Chang 4 + 5 reservoir in Luo 21 area.

Sample	Well	Horizon	Pore volume/cm^3^	Porosity/%	Liquid permeability/mD	Skewness	Sorting coefficient
L1	L21	Chang 4 + 5_2_^2^	1.49	5.38	0.10	0.45	1.63
L2	L255	Chang 4 + 5_1_^2^	2.82	11.17	0.42	0.49	2.10
L3	L115-100	Chang 4 + 5_2_^1^	0.47	1.82	0.05	0.07	1.56
L4	L115-100	Chang 4 + 5_2_^2^	1.63	6.04	0.26	0.37	2.50
Average			1.60	6.10	0.21	0.34	1.95

Sample	Well	Horizon	Median pressure/Mpa	Median radius/mm	Displacement pressure/Mpa	Maximum S_hg_/%	The mercury withdrawal efficiency/%
L1	L21	Chang 4 + 5_2_^2^	26.22	0.03	1.84	64.27	16.68
L2	L255	Chang 4 + 5_1_^2^	2.12	0.35	0.33	81.14	25.81
L3	L115-100	Chang 4 + 5_2_^1^	106.50	0.01	1.19	51.07	24.77
L4	L115-100	Chang 4 + 5_2_^2^	12.82	0.06	0.60	74.70	22.11
Average			36.91	0.11	0.99	67.79	22.34

#### Pore structure characteristics

**Figure 12:**
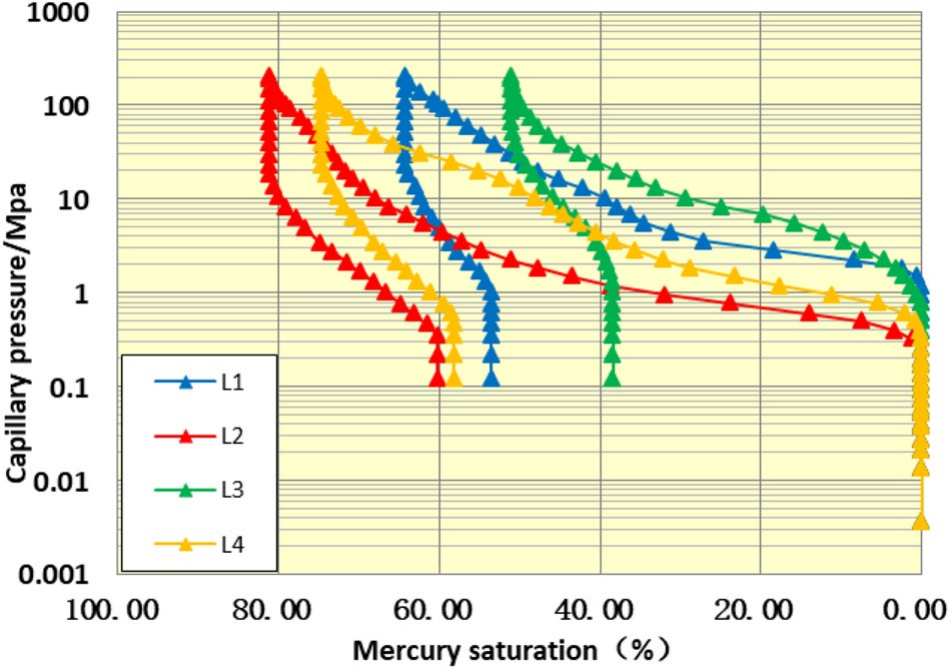
Capillary pressure curve of Chang 4 + 5 reservoir in Luo 21 area.

**Figure 13:**
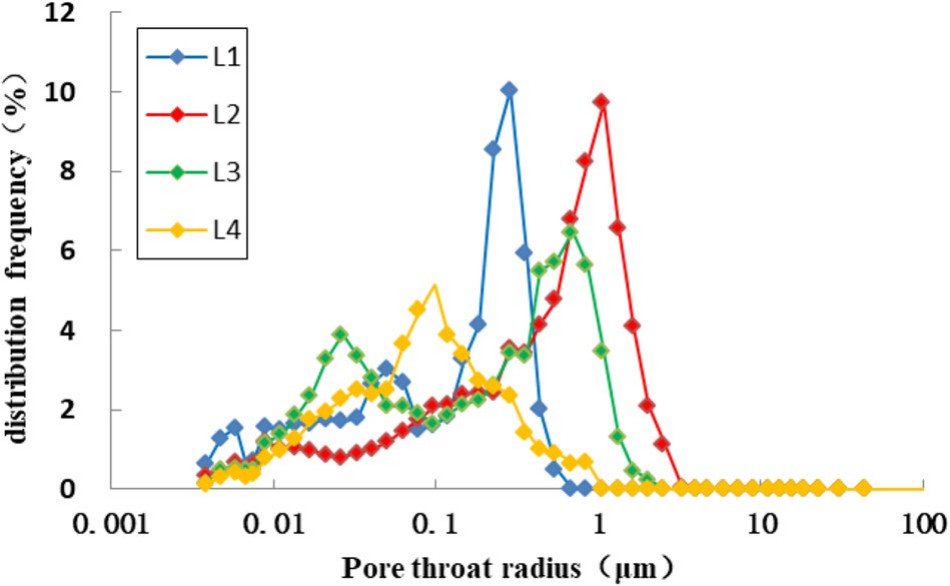
Pore throat radius of Chang 4 + 5 reservoir in Luo 21 area.

Through high pressure mercury injection experiment, the pore structure parameters of reservoir rocks (displacement pressure, median pressure, variation coefficient, sorting coefficient, uniformity coefficient, skewness, the maximal radii of pore throats, median radius, the maximum mercury saturation and the efficiency of mercury withdrawal) can be obtained, respectively^29–31^. From the parameters of capillary pressure curve (Table 1, Figs. 12, 13), it can be seen that the average displacement pressure of 4 samples from Chang 4 + 5 of Yanchang formation in the study area is higher, the median pressure is higher, the median radius is medium, the sorting coefficient is medium, the pore throat skewness is positive and coarse, the mercury saturation is higher, and the mercury withdrawal efficiency is low. The mercury injection curve has a certain platform, which appears in the finer throat. It can be judged that the capillary pressure curve is mainly low displacement pressure-thin throat type. Based on the analysis, it is concluded that the pore structure of the target section in the study area is well, the pore-throat connectivity is relatively well, however the oil displacement efficiency is poor.

## Data and experimental measurements

### Characteristics of water-flooding in plane

The characteristics of plane water-flooding in Luo 21 area are complex, and the sweep efficiency of water-flooding is low. Based on the statistics of water injection effectiveness of 15 injection-production well groups in Luo 21 area of Jiyuan Oilfield, according to the actual effect, the effective characteristics of oil wells are divided into three categories: Type I: after the effect, the productivity of single well increased significantly, and the water cut changed little. Type II: the characteristic of increasing production is not obvious, but the production capacity changes from decreasing to basically stable, and the water-cut increases slowly. Type III: after the effect, the water-cut increased obviously, basically entered the exploitation period of high water cut (water cut > 60%).

**Figure 14:**
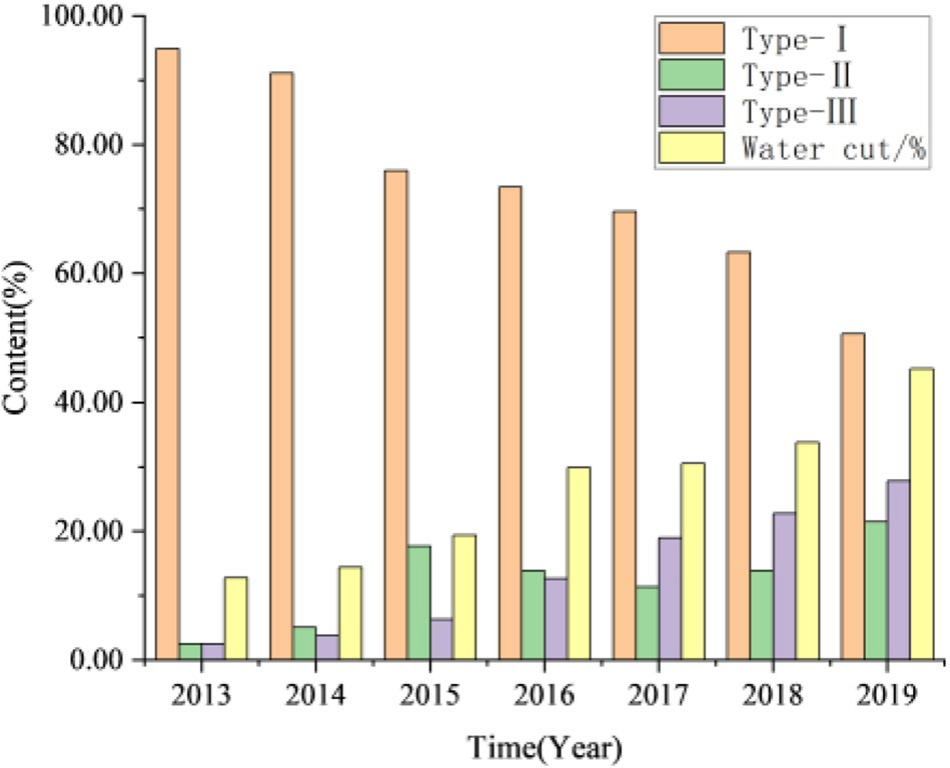
Statistics of effective types of Chang 4 + 5 in Jiyuan Oilfield, Ordos Basin.

There are 79 effective oil wells and the degree of effectiveness is 95.2%. Through the analysis and statistics of the production data of 83 oil wells in the study area, the effective types are classified, and the results are shown in Fig. 14. During the period from 2013 to 2014, when the oil field was put into production, the effective type of oil wells is mainly type I, accounting for more than 80% of the total number of wells. There are few effective oil wells of type III, and the water cut in the block is kept at a low level. From 2015 to 2017, during continuous water injection to replenish formation energy, the non-piston water-flooding is serious. The proportion of type I oil wells decreases rapidly and is transformed into type II oil wells, at the same time, the number of type III oil wells increases, which leads to the increase of water cut in the oil field. From 2018 to 2019, the proportion of the total number of type I oil wells decreased. The percentage of type III effective oil wells was 27.85%, and the water cut of the oil field was 45.17%.

**Figure 15:**
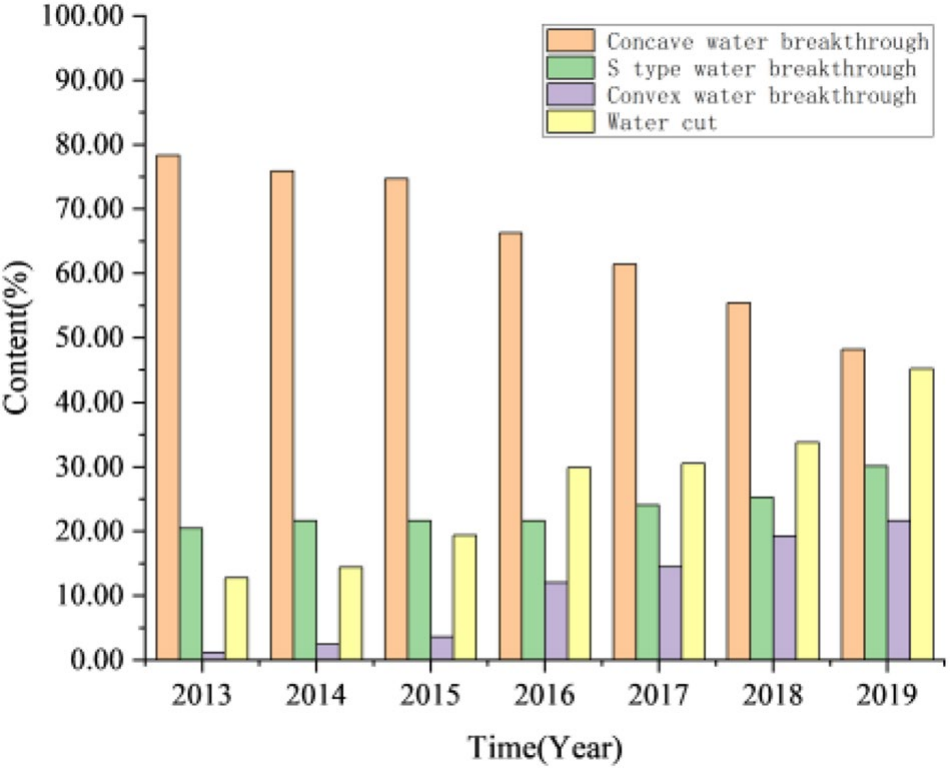
Statistics of water breakthrough in Chang 4 + 5 in Jiyuan Oilfield, Ordos Basin.

As of August 2019, there were about 83 water breakthrough oil well (water-cut > 10%) in the study area of Jiyuan Oilfield. From the perspective of reservoir development performance, the performance of water breakthrough oil wells can be divided into three types: convex water breakthrough (water cut growth rate decreases with time), S type water breakthrough (transitional type) and concave water breakthrough (water cut growth rate increases with time). The water breakthrough oil wells are classified, as shown in Fig. 15. During the period from 2013 to 2015, when the oil field was put into production, the percentage content of concave water breakthrough oil wells was more than 80%, that of convex water breakthrough oil wells was less than 20%, and the water cut of the oil field was kept at a relatively low level. From 2016 to 2018, the proportion of concave oil wells began to decrease, gradually changed to S type, and the water cut increased slowly. In 2019, S-type wells and convex water breakthrough wells reached the maximum, with 18 convex wells, accounting for 21.69%, 25 S type wells, accounting for 30.12%, 40 concave wells, accounting for 48.19%, and water cut of oil field is 45.17%.

### Characteristics of water-flooding in profile

The reservoir of Jiyuan Oilfield in Ordos Basin is mainly composed of small sand body complexs, and interlayer is developed between layers, which leads to poor physical properties of the reservoir. At the same time, the intrastratal and interlayer heterogeneity of reservoir is strong due to the physical properties in the longitudinal direction, pore structure characteristics, the changes of reservoir oiliness, the difference of the accumulation mode and shape of detrital minerals and the uneven distribution of minerals, resulting in uneven water injection profile of water injection wells^32^. The microfractures in Luo 21 area are well developed, the profile heterogeneity is strong, and the reserves producing degree of water-flooding is low. Intralayer: the injected water is mainly driven along the high permeability section, the reserves producing degree of the low permeability section is low, and the remaining oil is enriched. Interlayer: the difference between layers is large, the reserves producing degree of layers is uneven, and the problem between layers is complex.

**Figure 16:**
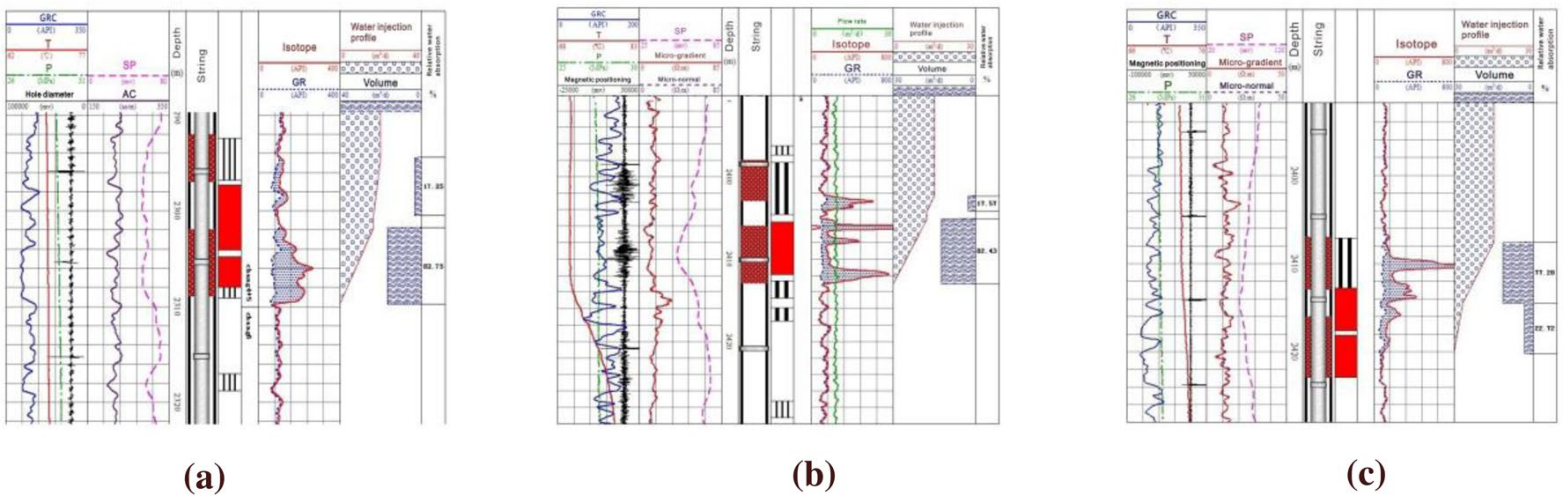
Analysis of prosodic characteristics of coring well. (**a**) Uniform absorption; (**b**) finger absorption; (**c**) peak absorption.

**Figure 17:**
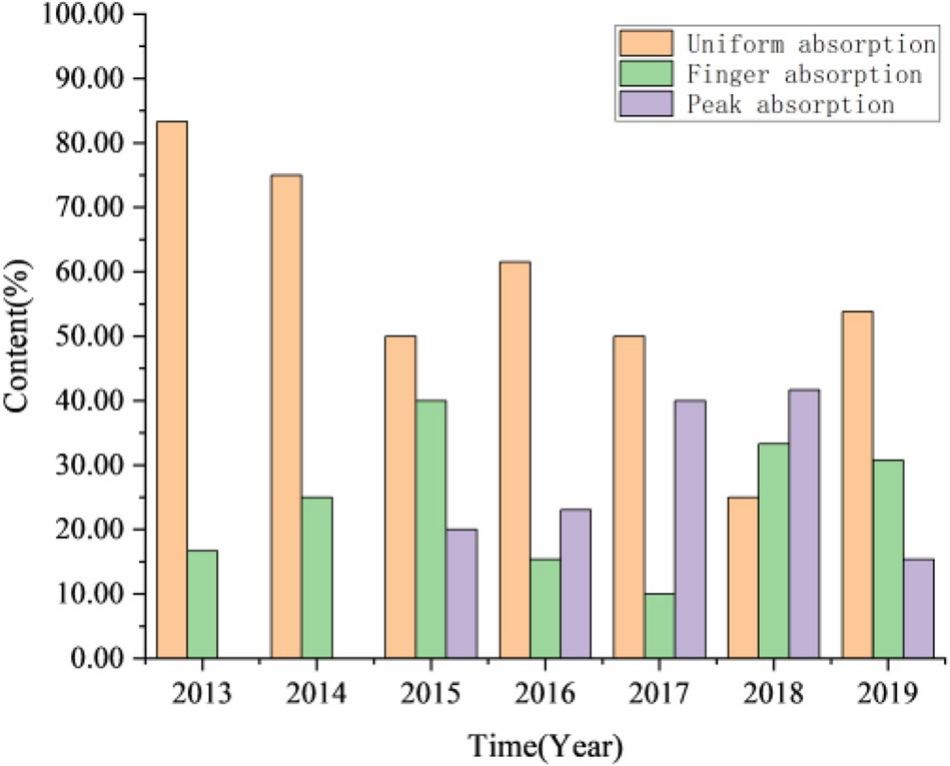
Statistics of Chang 4 + 5 Water absorption types in Jiyuan Oilfield, Ordos Basin.

According to the water absorption pattern, the water injection profile of the injection well is divided into three types (Fig. 16): uniform absorption, finger absorption and peak absorption. Through the statistics of the water absorption profile over the years, it can be seen from Fig. 17 that at the initial stage of development, the production situation of the study area is relatively simple, mainly including uniform water absorption and finger water absorption. From 2013 to 2019, the proportion of uniform absorption remained relatively stable. By 2015, due to intrastratal and interlayer heterogeneity and water injection development, peak absorption appeared in some layers. In 2018, the ratio of finger absorption and peak absorption is more than uniform, and the water absorption profile becomes more complex, which reduces the development efficiency.

## Results

Microscopically, reservoir of Chang 4 + 5 in Jiyuan oilfield has great changes in lithology and physical properties, complex pore structure and strong heterogeneity, short oil recovery period without water and rapid increase of water cut in the process of water-flooding. Macroscopically, the heterogeneity and wettability of the reservoir have a decisive effect in the percolation law of the fluid, thus affecting the recovery of the low permeability reservoir.

### Sedimentary environment

#### In the plane

The sedimentary environment is changeable, which makes the change law of the reservoir in the plane complicated. The results of plane water-flooding characteristics show that type I effective wells are basically concentrated in the areas with thicker sand bodies, better connectivity and relatively good reservoir physical properties. The sand bodies in the main study area of type II effective oil wells are distributed in strips in the middle of the river. Type III effective wells are mainly distributed in the southwest and northeast edge of the study area where the sand bodies are thin, the reservoir physical properties are poor and fractures are developed. Thin sand body thickness, uneven distribution of physical properties between wells, and low porosity and low permeability play a role in hindering the energy transmission of water injection.

Chang 4 + 5 reservoir in Jiyuan Oilfield belongs to low permeability reservoir^2^. Due to the directional strengthening of fracture to permeability after fracturing, the percolation mechanism of the reservoir is obviously changed in the process of production, resulting in watering out and even flooding. Due to the existence of natural microfractures, artificial fractures or high permeability zones, the injected water advances unidirectionally along the direction of low resistance, and the horizontal and vertical heterogeneity of the reservoir is intensified, resulting in the rapid rise of water cut in the production wells connected with the fractures. however, the production wells with lateral fractures are not easy to be effective. The direction of water breakthrough is consistent with the direction of principal stress, the oil well is easily flooded, and the effective period is short. The water breakthrough is characterized by a sharp increase in liquid content, a rapid increase in watering cut and a large decrease in productivity. The inverted nine-spot rhombus injection-production pattern is adopted in Jiyuan oilfield, and the well row direction is consistent with the fracture distribution direction, which is 75° in the northeast. In the fracture development zone, water can be seen quickly in corner wells and slowly in side wells.

#### In the longitudinal direction

The typical low permeability reservoir in Chang 4 + 5 reservoir of Jiyuan Oilfield is delta front deposits, and the main micro facies developed are mouth bar, subaqueous distributary channel and interdistributary estuary. The sedimentary micro facies also has a certain influence on the water injection profile, so the comparison method between the spontaneous potential of the water injection profile and the morphology of natural gamma and water absorption types is useful. According to the statistics of the curve types of water injection profile, the uniform absorption is mainly bell-shaped and box-shaped, and the peak absorption is mainly bell-shaped.

**Figure 18:**
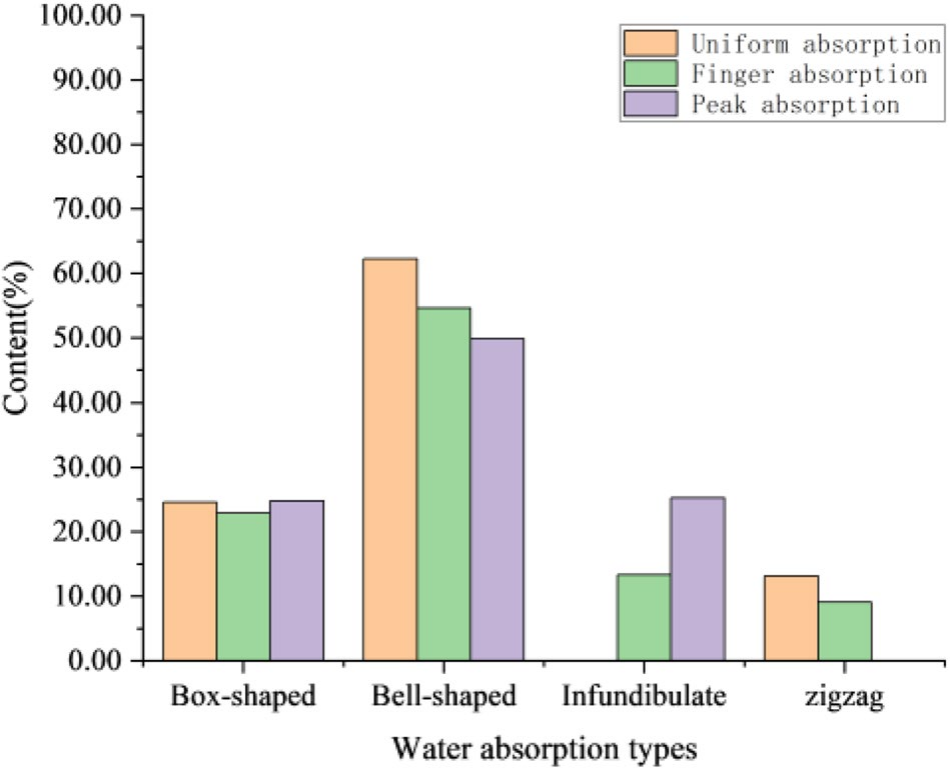
Proportion histogram of water absorption type.

Through the statistics of the curve types corresponding to 28 water injection profiles in 2013 and 2019, it can be seen from Fig. 18 that the curve types in the whole region are mainly box-shaped and bell-shaped, in which the proportion of bell-shaped and box-shaped in finger absorption is higher, the proportion of finger type and infundibulate is higher in peak absorption, and the uniform water absorption is mainly bell-shaped, so the sedimentary micro-facies has a certain influence on the type of water absorption.

### Reservoir heterogeneity

#### Plane heterogeneity

The formation of oil and gas reservoir is affected by sedimentary environment, diagenesis and tectonics and artificially induced changes in the later stage, resulting in uneven changes in the spatial distribution and internal attributes of the reservoir. Based on the analysis of the physical data of the plane heterogeneity of the main layer in the study area (Table 2), the relatively high porosity and high permeability area is widely distributed in the center of the main channel and the north of the study area. The porosity of the high porosity zone is more than 8%, and the local porosity is more than 15.1%; the permeability of the high permeability zone is more than 5 mD, and the local permeability is greater than 20 mD. It can be seen that there are obvious differences in reservoir physical properties among the three types of water absorption profiles. Among them, the permeability range of uniform water absorption reservoir is 36.63, and the permeability mutation coefficient is 1.8, which is a homogeneous formation. The permeability range of finger water absorption reservoir is 93.00, the mutation coefficient of permeability is 2.6, and the heterogeneity is moderate. The location permeability range of the peak water absorption reservoir is 1700.00, the permeability mutation coefficient is 4.2, and the heterogeneity is strong.

**Table 2:** Plane distribution of physical parameters of oil reservoir.

Water absorption type	Porosity/%	Permeability/10^-3^µm^2^	Permeability range
Uniform absorption	5.2–15.5	1.6–60.2	37.63
Finger absorption	2.7–20.6	0.9–83.7	93.00
Peak absorption	1.5–27.1	0.5–850	1700.00

Water absorption type	Permeability mutation coefficient	Heterogeneity	Displacement efficiency/%
Uniform absorption	1.8	Homogeneous	58.56
Finger absorption	2.6	Moderate	39.21
Peak absorption	4.2	Strong	21.27

#### In-layer heterogeneity

Intra-layer heterogeneity is the internal cause of intra-layer contradiction in production, which directly controls the watered out thickness and sweep efficiency in single sand layer. Statistics show that (Table 3), the maximum range of Chang 4 + 5_2_^1^ reservoir in Luo 21 area of Jiyuan oilfield is 270.5 and the minimum is 1.63, the maximum mutation coefficient is 7.19 and the minimum is 1.89, the maximum variation coefficient is 1.35 and the minimum is 0.46, and the heterogeneity is weak. The maximum range of Chang 4 + 5_2_^2^ reservoir is 1700.41 and the minimum is 3.21, the maximum mutation coefficient is 2.31 and the minimum is 1.45, the maximum variation coefficient is 1.7 and the minimum is 0.36, and the heterogeneity is strong. Generally speaking, the heterogeneity of Chang 4 + 5 layer is moderate-strong heterogeneity (Table 4).

**Table 3:** Permeability heterogeneity in the block.

Block	Horizon	Range		Mutation coefficient		Variation coefficient	
		Max	Min	Max	Min	Max	Min
	Chang4 + 5_2_^1^	270.5	1.63	7.19	1.89	1.35	0.46
	Chang4 + 5_2_^2^	1700.41	3.21	2.31	1.45	1.7	0.38

**Table 4:** Interlayer parameters in the block.

Block	Horizon	Interlayer distribution frequency (bar/m)		Interlayer distribution density/%	
		Max	Min	Max	Min
	Chang4 + 5_2_^1^	0.82	0.17	0.62	0.25
	Chang4 + 5_2_^2^	0.28	0.14	0.59	0.23

#### Interlayer heterogeneity

The parameters of range, mutation coefficient and variation coefficient of interlayer permeability can reflect the heterogeneity of interlayer permeability. Analysis of physical property data of Chang4 + 5_2_^1^, Chang4 + 5_2_^2^ in Luo 21 area of Jiyuan Oilfield. The results show that (Table 5), the maximum permeability range of Chang 4 + 5_1_ reservoir is 66.8 and the minimum is 2.32, the maximum mutation coefficient is 3.85 and the minimum is 1.31, and the maximum variation coefficient is 66.8 and the minimum is 2.32 in Luo 21 area. The maximum permeability range of Chang 4 + 5_2_ reservoir is 213.76 and the minimum is 2.17, the maximum mutation coefficient is 4.56 and the minimum is 1.31, and the maximum variation coefficient is 1.8 and the minimum is 0.26 in Luo 21 area. Comprehensive evaluation: the interlayer heterogeneity in the study area is in a medium-strong degree.

**Table 5:** Interlayer permeability heterogeneity in the block.

Block	Horizon	Range		Mutation coefficient		Variation coefficient	
		Max	Min	Max	Min	Max	Min
L21	Chang4 + 5_1_	66.8	2.32	3.85	1.31	0.74	0.28
	Chang4 + 5_2_	213.76	2.17	4.56	1.31	1.8	0.26

The interlayer interval can reflect the interlayer heterogeneity of the reservoir, and it can block the fluid movement. It is found that Chang 4 + 5 in Luo 21 area is mainly mudstone, and the average thickness of the interlayer is 6.8 m. It has a good blocking effect. It is not easy to fracture, so it can really block the movement of fluid (Table 6).

**Table 6:** Restraining barrier of Chang 4 + 5 in the block.

Horizon	Thickness (m)		Wells		
	Max	Min	Thickness = 0	Total	Proportion/%
Chang4 + 5_2_^1^-Chang4 + 5_2_^2^	14.6	0.48	9	103	8.74
Chang4 + 5_2_^2^-Chang4 + 5_2_^3^	25.94	0.55	–	79	–

### Microscopic pore structure

The key factor affecting the water-flooding efficiency of low permeability reservoir is the reservoir microscopic pore structure. Combined with mercury injection data, casting thin sections and production performance, the influence of pore types on reservoir water flooding development is analyzed (Table 7).

**Table 7:** Characteristics of porosity and water flooding in the study area.

Pore category	Pore characteristics	Water-flooding characteristics
High porosity and permeability	The grain sorting is good, the pore throat is uniformly distributed, and the connection between the pore and the throat is reticulate	It is usually dominated by uniform displacement, and the injected water sweep efficiency are high
Medium porosity and low permeability	The grain sorting is poor, the distribution of pore throat is uneven, and the type of throat is mainly flaky	It is usually dominated by peak displacement, the injected water sweep efficiency are low, and the water cut increases rapidly
Low porosity and low permeability	The grain sorting is relatively good, the pore throat is uniformly distributed, and the connection between the pore and the throat is a thin mesh	It is usually dominated by finger displacement, the injection water sweep efficiency are medium, and the displacement efficiency is relative good

### Wettability

**Figure 19:**
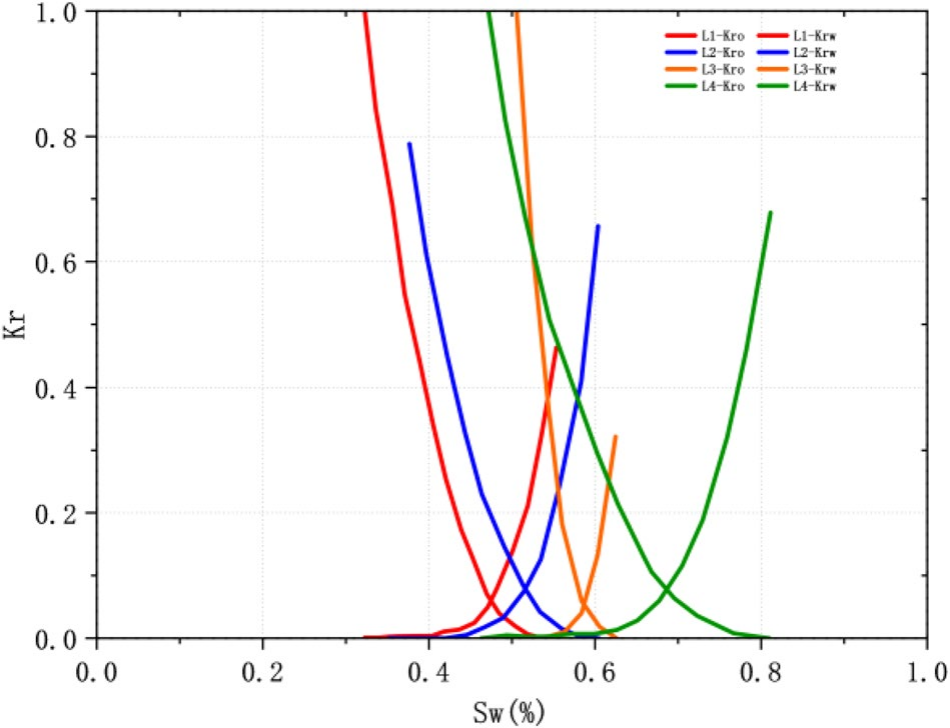
Relative permeability curve of Chang 4 + 5 reservoir in the study area.

In the process of oil and gas exploitation, the wettability of reservoir rock directly affects the flow law and distribution of oil and water, thus affecting the oil recovery^33,34^. Through the test of 4 samples in Luo 21 area (Table 8, Fig. 19), the relative permeability curve of Chang 4 + 5 reservoir in the study area is divided into three types, and the oil displacement efficiency of lipophilic reservoir is the lowest, which is 33.64%. The average oil displacement efficiency of neutral wetting reservoirs is 51.27%, and the oil displacement efficiency of hydrophilic reservoirs is the highest (64.27%).

**Table 8:** Relative permeability data of Luo 21 area in Jiyuan Oilfield.

Number	Well	Porosity/%	Permeability/10^-3^μm^2^	Swi%	Sw (Kro = Krw) /%	1-Sor/%	Wettability	Displacement efficiency/%
L-1	L21	5.38	0.54	32.29	46.93	55.07	Lipophilicity	33.64
L-2	L255	11.17	1.12	35.19	51.21	60.36	Neutrality	48.84
L-3	L115-100	1.82	0.36	50.54	58.35	62.26	Neutrality	53.70
L-4	L115-100	6.04	0.98	46.37	67.58	80.84	Hydrophilic	64.27

## Discussions

In the process of water-flooding, there are many factors that control the water flooded layer, including the internal factors of the reservoir and the conditions of water injection. The internal factors of the reservoir include porosity, permeability, shale mass fraction and flow unit type. The conditions of water injection include the time of water injection, the properties of water injection, the volume of water injection and the pattern of well pattern, etc. Taking Chang 4 + 5_2_^1^ and Chang 4 + 5_2_^2^ in Luo 21 area of Jiyuan Oilfield as examples, the water-flooding law of low permeability reservoir is summarized by comprehensively considering reservoir internal factors and water injection factors.

**Figure 20:**
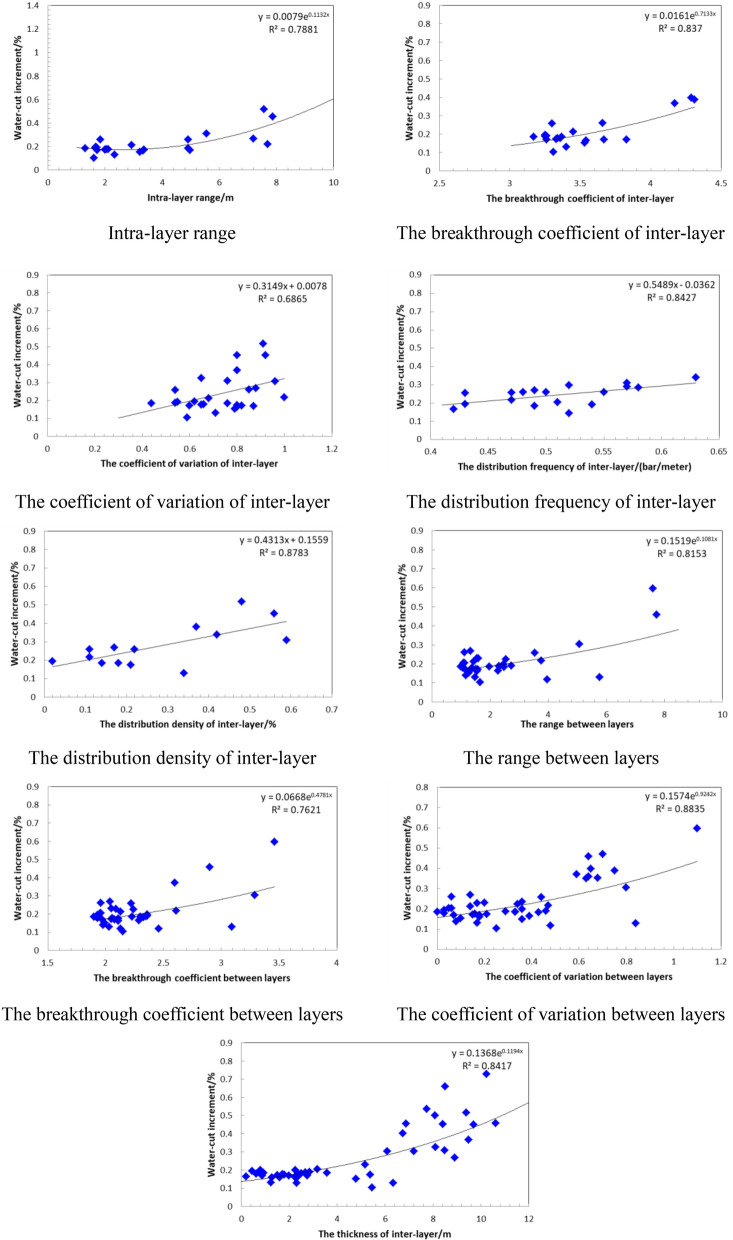
The relationship between the effective reservoir parameters and Water-cut increment.

**Figure 21:**
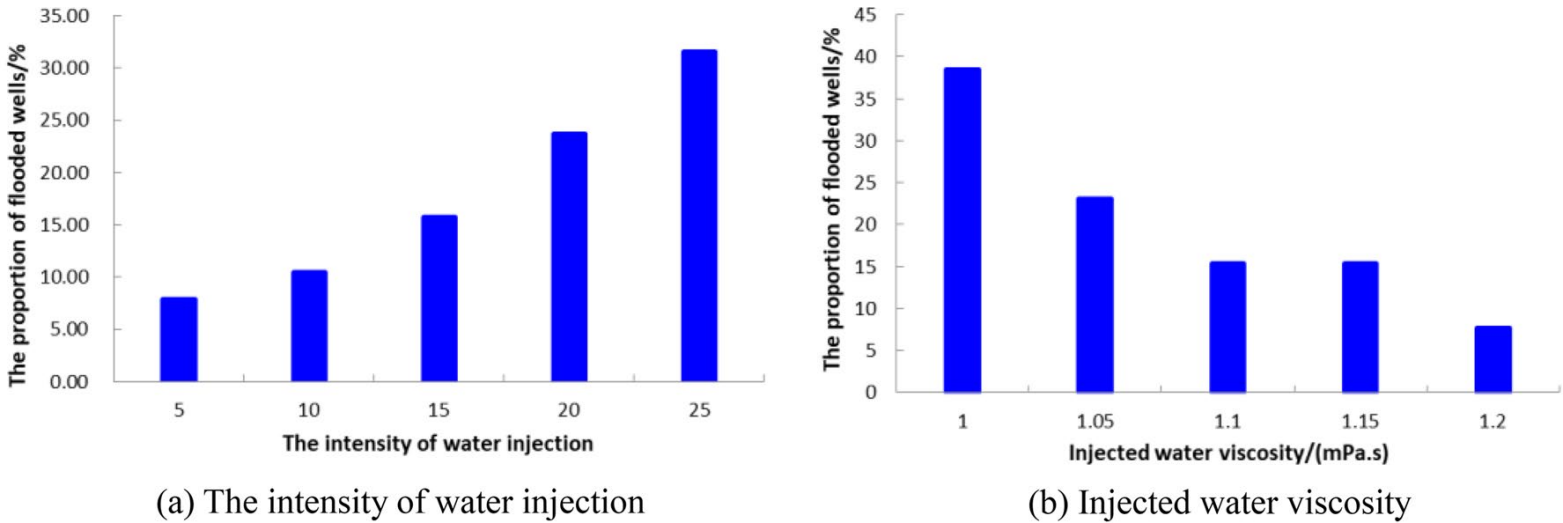
The relationship between the injection condition and water-flooding.

1. Through the combination of reservoir static parameters and development dynamic parameters, the relationship between reservoir physical parameters and water cut increment is drawn respectively (Fig. 20). It can be seen that when the intra-layer range is greater than 4.65, the breakthrough coefficient is greater than 3.54, the coefficient of variation is greater than 0.7, the distribution frequency of inter-layer is greater than 0.5 per meter, and the distribution density is greater than 0.435%, the range between layers is greater than 6.86, the breakthrough coefficient is greater than 2.58, the coefficient of variation is greater than 0.51, and the thickness of inter-layer is greater than 7.54 m, the increment of reservoir water cut increases, which leads to the weakening of oil displacement efficiency.
2. Under the same conditions of water injection, the internal factors of the reservoir have a great influence on the watered-out reservoir. When the heterogeneity of the reservoir is strong, the injected water is easy to percolate along the high permeability channel with less resistance, resulting in finger shape and peak shape in the water injection profile, that is, the reservoir in the high permeability zone has a high degree of water-flooding (see Supplementary Information 1).
3. Under the same condition of internal factors in the reservoir, the water out rate is proportional to the intensity of water injection and inversely proportional to the viscosity of injected water (Fig. 21), that is, the injection condition is the main factor affecting water-flooding.
4. In order to improve the development efficiency of the reservoir, according to the characteristics of water-flooding, combined with mercury injection displacement parameters and microscopic pore structure parameters, the reservoir is divided into three types. The classification of reservoir types is shown in Table 9. It can be seen that the microscopic pore structure parameters of different reservoir types are quite different, and the watered-out law also shows different characteristics. Under the condition of strong water-flooding, type I reservoir is generally moderately watered out and type II reservoir is generally weakly watered out. Type III reservoir is usually the main area where remaining oil is enriched during development.

**Table 9:** Table reservoir classification of conglomerate reservoir.

Reservoir types	Mercury injection parameters			Microcosmic pore structure parameters					
	Maximum S_Hg_/%	The mercury withdrawal efficiency/%	Displacement pressure/MPa	Median radius/µm	Median pressure/MPa	Sorting coefficient	Skewness	Porosity/%	Permeability/mD
Types I	$\frac{69.485}{{64.28\sim 74.70}}$	$\frac{19.395}{{16.68\sim 22.11}}$	$\frac{1.22}{{0.60\sim 1.84}}$	$\frac{0.045}{{0.03\sim 0.06}}$	$\frac{19.52}{{12.82\sim 26.22}}$	$\frac{2.065}{{1.63\sim 2}}$	$\frac{0.41}{{0.37\sim 0.45}}$	$\frac{5.71}{{5.38\sim 6.04}}$	$\frac{0.18}{{0.10\sim 0.26}}$
Types II	$\frac{62.885}{{51.07\sim 74.70}}$	$\frac{23.44}{{22.11\sim 24.77}}$	$\frac{0.895}{{0.60\sim 1.19}}$	$\frac{0.035}{{0.01\sim 0.06}}$	$\frac{59.66}{{12.82\sim 106.5}}$	$\frac{2.03}{{1.56\sim 2.50}}$	$\frac{0.22}{{0.07\sim 0.37}}$	$\frac{3.93}{{1.82\sim 6.04}}$	$\frac{0.155}{{0.05\sim 0.26}}$
Types III	$\frac{66.1055}{{1.07\sim 81.14}}$	$\frac{25.29}{{24.77\sim 25.81}}$	$\frac{0.76}{{0.33\sim 1.19}}$	$\frac{0.18}{{0.01\sim 0.35}}$	$\frac{54.31}{{2.12\sim 106.5}}$	$\frac{1.83}{{1.56\sim 2.10}}$	$\frac{0.28}{{0.07\sim 0.49}}$	$\frac{{{6}.{495}}}{{{1}.{82}\sim {11}.{17}}}$	$\frac{0.235}{{0.05\sim 0.42}}$

$$\frac{{{\text{Average}}}}{{{{\text{Minimum}\sim \text{Maximum}}}}}$$

## Conclusion

1. The lithology of Chang 4 + 5 reservoir in Jiyuan Oilfield is mainly gray fine-grained debris-feldspar sandstone, containing a small amount of feldspathic sandstone and feldspathic lithic sandstone. The pore types are mainly intergranular pore (1.9%) and feldspar dissolution pore (1.0%), and the cementation type is mainly enlarged-pore type. The cementation type is mainly pore-cementation, and the support type is mainly grain-supported. The capillary pressure curve is mainly low displacement pressure-thin throat type. The common sedimentary structures in the study area mainly include deformation structure, bedding plane structure, bedding structure and biogenic structure
2. The oil displacement efficiency of Chang 4 + 5 reservoir in Jiyuan oilfield is medium. In the process of water-flooding, the microscopic seepage paths of chang 4 + 5 reservoir include uniform displacement, finger displacement and peak displacement, and their correspondent oil displacement efficiency reduces in turn under the same conditions. The characteristics of plane water-flooding in the study area are complex, and the sweep efficiency of water flooding is low. The plane effect is mainly the increase of type I effect and the stability of type II effect, and the local high permeability has type III effect. The water cut in the plane is mainly convex type and S type, and the water cut in the oil field is in the middle level.
3. Pore throat structure is a key parameter affecting water-flooding seepage law and oil displacement efficiency, and the efficiency of water-flooding is affected by the distribution form and size of throat. The distribution of reservoir physical properties, reservoir heterogeneity and pore structure are all controlled by diagenesis, and their effects on water-flooding efficiency are consistent. Rock wettability directly affects the displacement path and seepage law of injected water, thus affecting the oil displacement efficiency.
4. Under the same conditions of water injection, when the heterogeneity of the reservoir is stronger, the degree of water-flooding is higher. Under the same internal factors of the reservoir, the water-flooding rate is proportional to the intensity of water injection and inversely proportional to the viscosity of injected water. The oil recovery data and the pore structure parameters should be emphasized in the process of Chang 4 + 5 reservoir exploitation in Jiyuan Oilfield, and priority should be given to reservoir with high permeability in designing reasonable exploitation techniques and procedures.

## Supplementary Information

Supplementary Information
