# Author Correction: Water-flooding characteristics of lithologic reservoir in Ordos basin

**DOI:** 10.1038/s41598-021-88763-x

**Published:** 2021-04-21

**Authors:** Jie He, Xiaodong Liu, Xinyu Zhu, Tao Jiang, Hui He, Lin Zhou, Qinghai Liu, Yushuang Zhu, Linyu Liu

**Affiliations:** 1 State Key Laboratory of Continental Dynamics, Department of Geology, Northwest University, Xi’an, 710069 China; 2 No. 7 Oil Production Plant, PetroChina Changqing Oilfield Company, Xifeng, 745000 Gansu China; 3 No. 1 Oil Production Plant, PetroChina Daqing Oilfield Company, Daqing, 163001 Heilongjiang China

Correction to: *Scientific Reports* 10.1038/s41598-021-82035-4, published online 28 January 2021

The original version of this Article contained an error in Affiliation 2, which was incorrectly given as ‘No. 11 Oil Production Plant, PetroChina Changqing Oilfeld Company, Xifeng 745000, Gansu, China’. The correct affiliation is listed below:

No. 7 Oil Production Plant, PetroChina Changqing Oilfield Company, Xifeng 745000, Gansu, China

This error has now been corrected in the HTML and PDF versions of the Article, and in the accompanying Supplemental Material.

