# Supplementary material for: Water-flooding characteristics of lithologic reservoir in Ordos basin
Source: Sci Rep. 2021 Jan 28;11:2503. doi: 10.1038/s41598-021-82035-4 (PMC7844225; doi:10.1038/s41598-021-82035-4)
Supplement: Supplementary file 1 — Supplementary Information [file 41598_2021_82035_MOESM1_ESM.docx]

**Water-flooding characteristics of lithologic reservoir in Ordos basin**

Jie He^a^, Xiaodong Liu^b^, Xinyu Zhu^c^, Tao Jiang^c^, Hui He^a^, Lin Zhou^a^, Qinghai Liu^a^, Yushuang Zhu^a^[[1]](#footnote-1)^^, Linyu Liu^a^

a. State Key Laboratory of Continental Dynamics/Department of Geology, Northwest University, Xi’an, 710069, China;

b. No. 7 Oil Production Plant，PetroChina Changqing Oilfield Company, Xifeng 745000, Gansu, China

c. No. 1 Oil Production Plant，PetroChina Daqing Oilfield Company, Daqing 163001, Heilongjiang, China;

As shown in the figure, the Fig. 1 is the well location of the study area; The stratigraphic division of the study area is carried out along the direction of the provenance and perpendicular to the provenance. The figure (Fig. 2,Fig. 3,Fig. 4) is the direction of the provenance, and the figure (Fig. 5,Fig. 6,Fig. 7) is the direction of the vertical provenance.


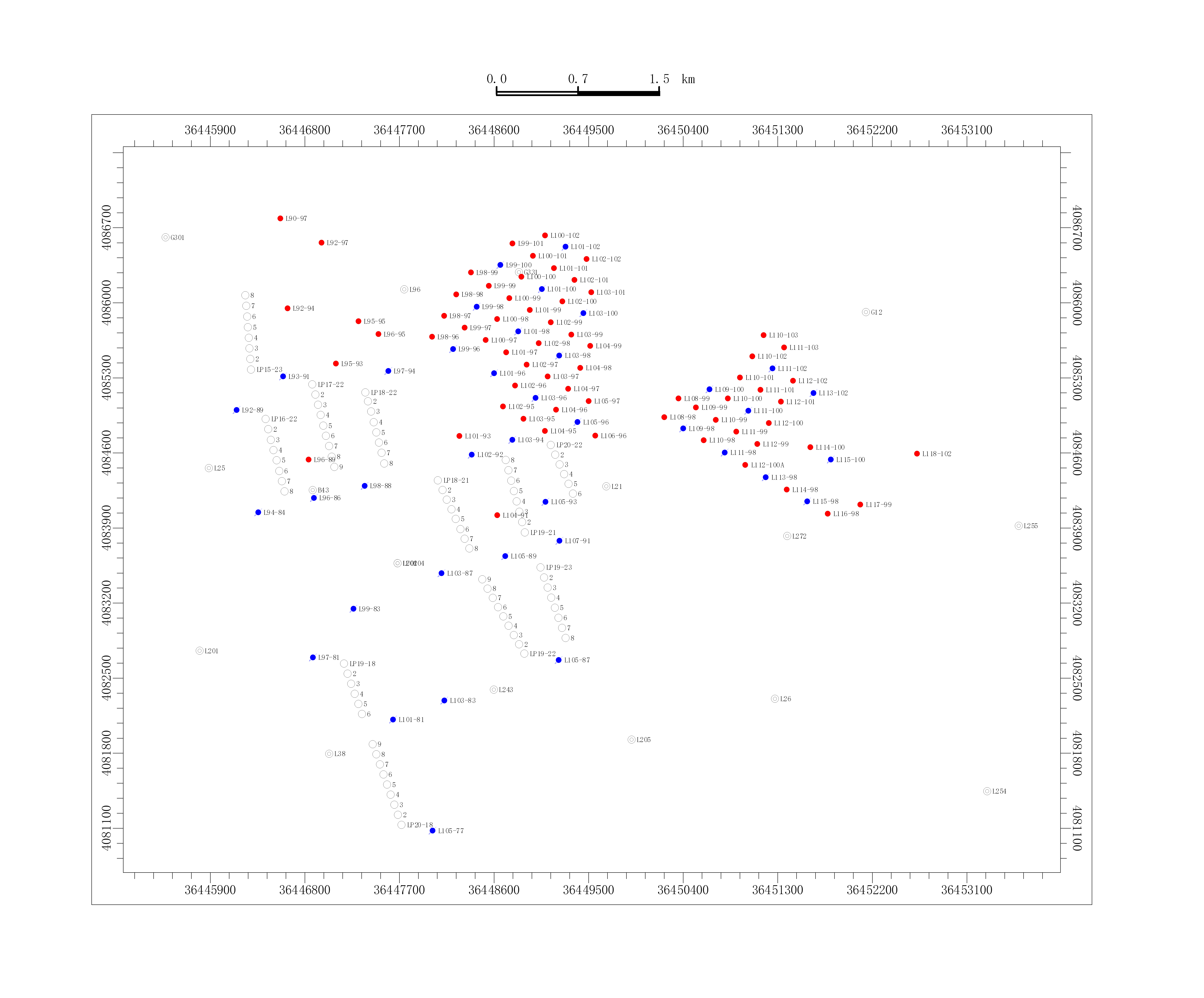


Fig. 1 Well location of the study area. Created using Gxplorer 5.8(http://www.11467.com/qiye/27642862.htm#gongshang)


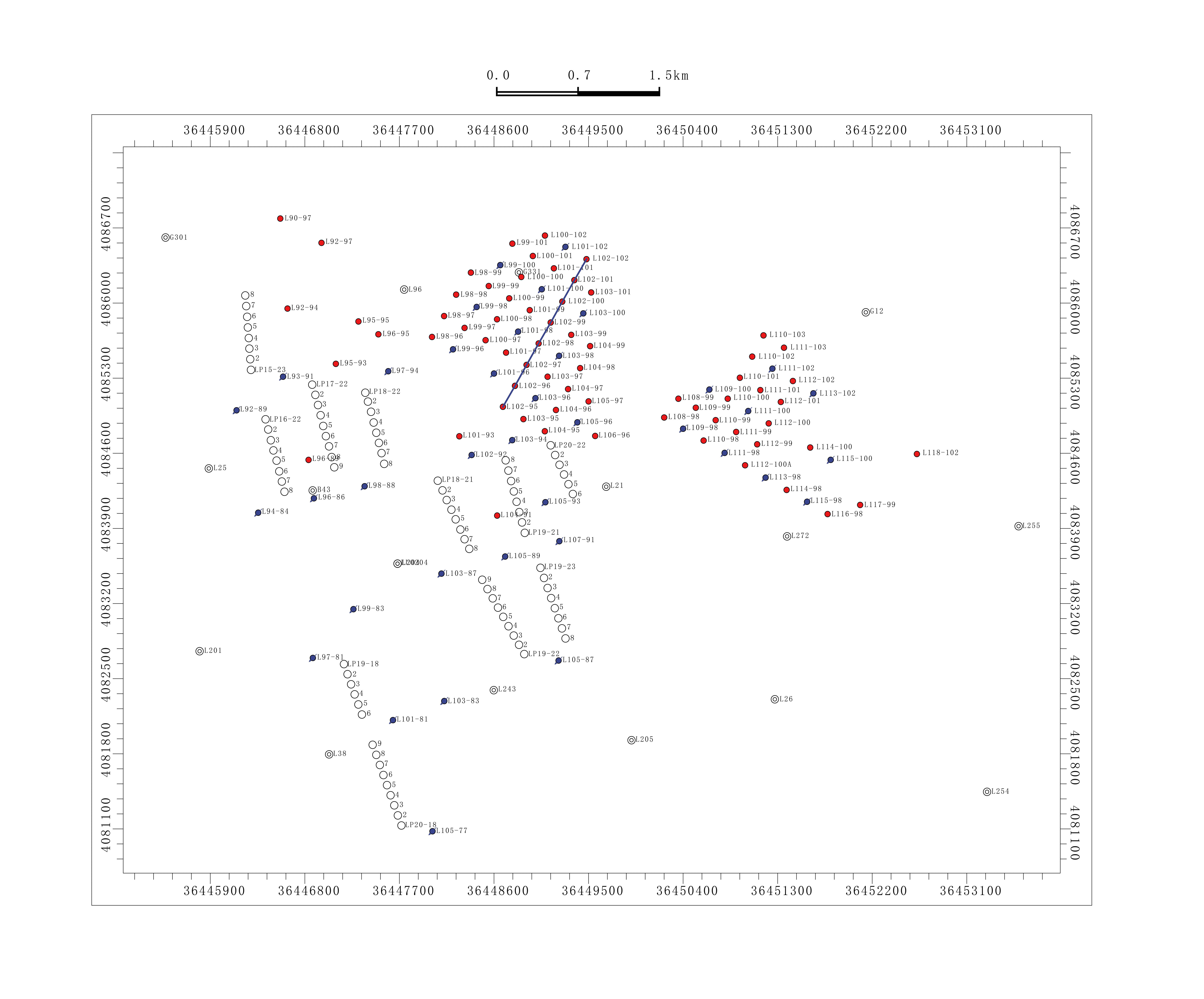


Fig. 2 Parallel to the direction of the provenance. Created using Gxplorer 5.8(http://www.11467.com/qiye/27642862.htm#gongshang)


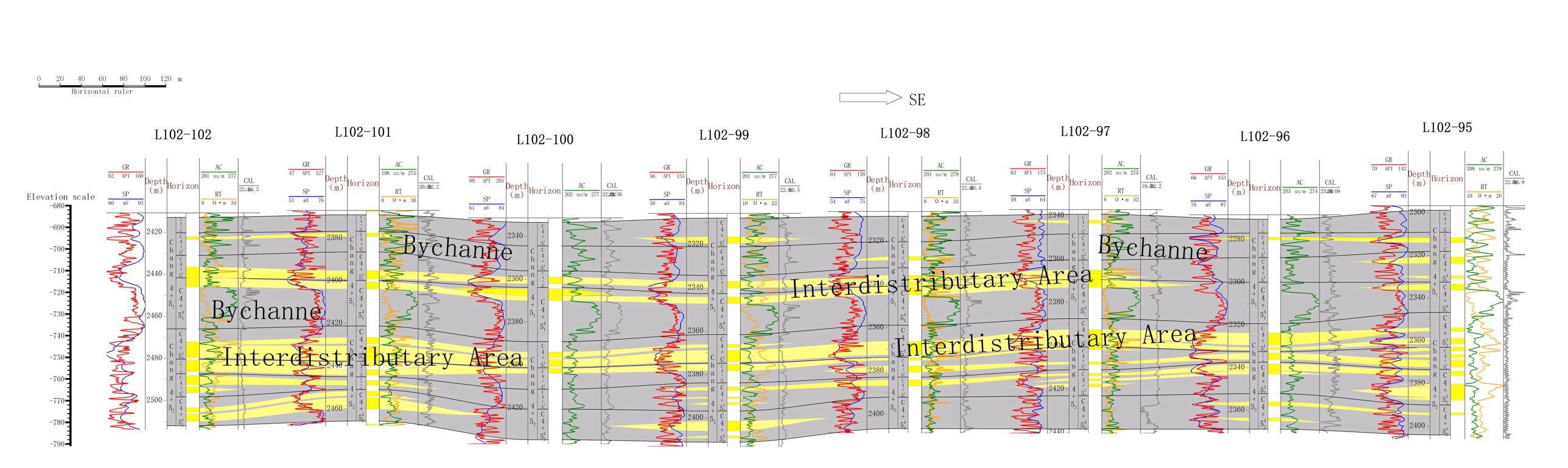


Fig. 3 Sedimentary facies. Created using Gxplorer 5.8(http://www.11467.com/qiye/27642862.htm#gongshang)


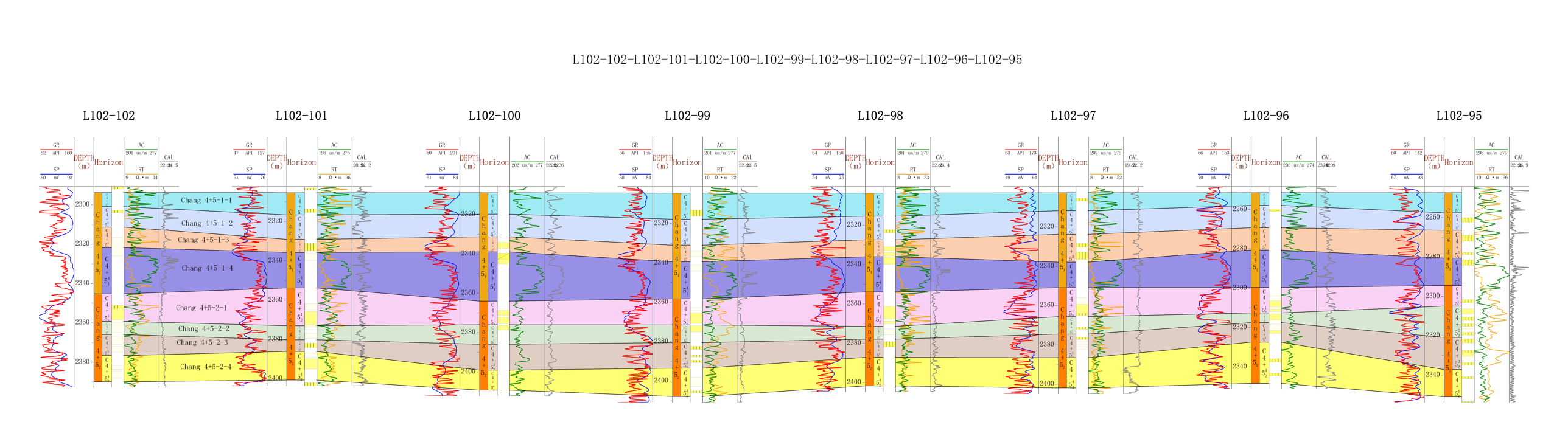


Fig. 4 Horizon of study area. Created using Gxplorer, 5.8(http://www.11467.com/qiye/27642862.htm#gongshang)


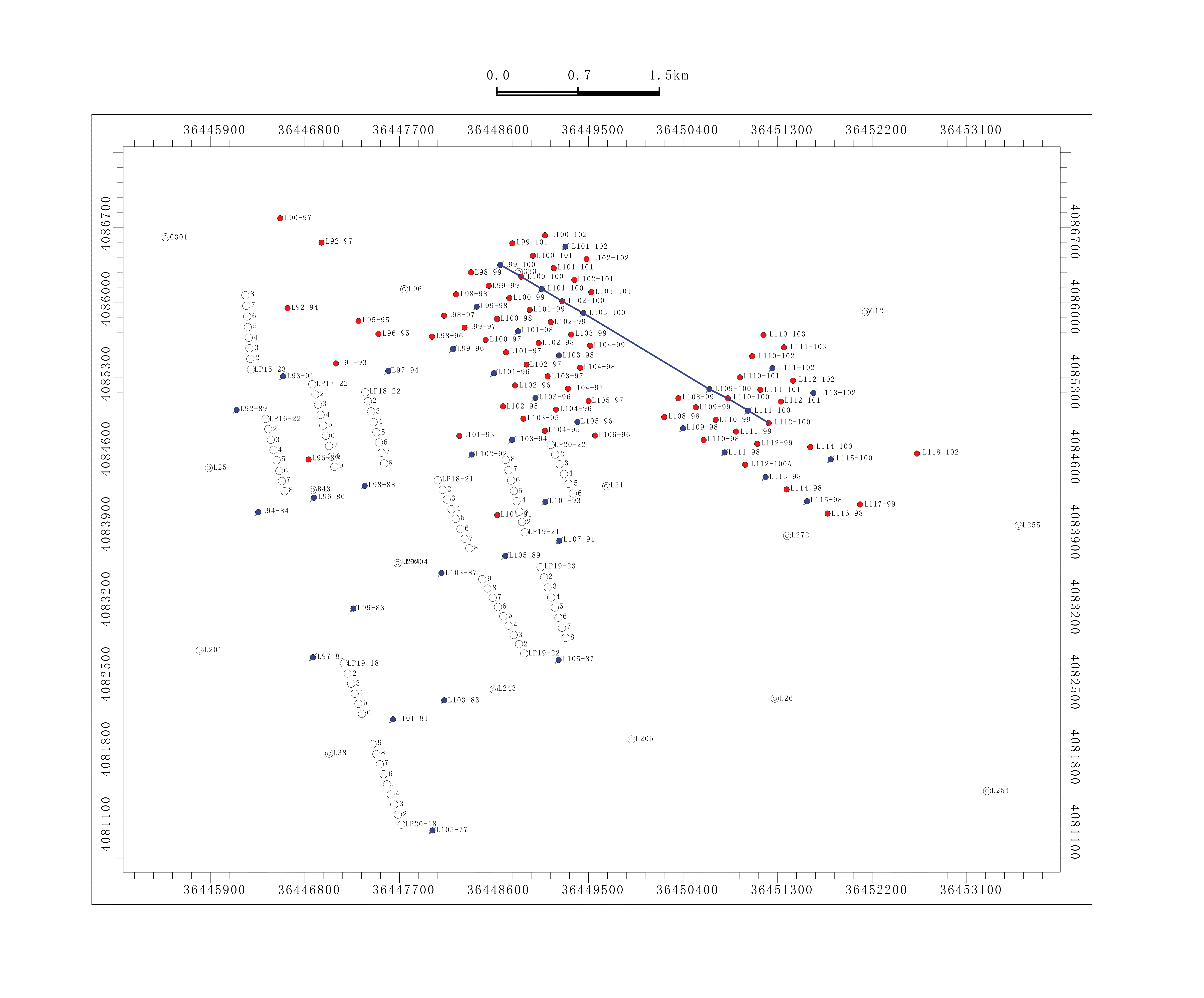


Fig. 5 Perpendicular to the direction of the provenance. Created using Gxplorer 5.8(http://www.11467.com/qiye/27642862.htm#gongshang)


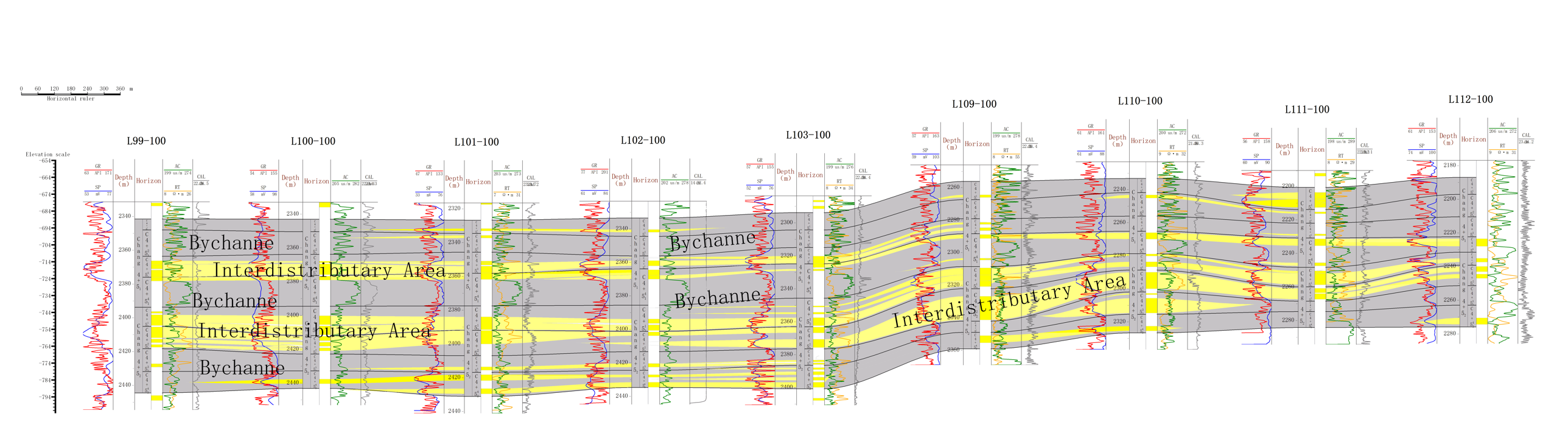


Fig. 6 Sedimentary facies. Created using Gxplorer 5.8(http://www.11467.com/qiye/27642862.htm#gongshang)





Fig. 7 Horizon of study area. Created using Gxplorer 5.8(http://www.11467.com/qiye/27642862.htm#gongshang)

Rock mineral composition (Table. 1)：

Table. 1 Rock mineral composition

| Felspar(%) | Cuttings(%) | Quartz(%) |
| --- | --- | --- |
| 16.233 | 56.564 | 27.204 |
| 10.189 | 54.546 | 35.266 |
| 12.531 | 46.028 | 41.441 |
| 13.741 | 37.793 | 48.466 |
| 25.012 | 33.331 | 41.657 |
| 27.357 | 36.083 | 36.560 |
| 20.326 | 40.298 | 39.375 |
| 23.067 | 40.305 | 36.628 |
| 26.731 | 40.086 | 33.183 |
| 29.559 | 40.322 | 30.119 |
| 31.412 | 42.844 | 25.744 |
| 33.880 | 44.595 | 21.525 |
| 35.510 | 47.603 | 16.887 |
| 32.187 | 55.346 | 12.467 |
| 29.547 | 54.338 | 16.115 |
| 29.034 | 51.076 | 19.890 |
| 27.484 | 52.588 | 19.928 |
| 24.505 | 53.810 | 21.685 |
| 24.641 | 51.579 | 23.779 |
| 24.655 | 48.833 | 26.511 |
| 22.680 | 50.831 | 26.489 |
| 26.804 | 44.577 | 28.619 |
| 21.158 | 49.568 | 29.274 |
| 19.399 | 53.053 | 27.548 |
| 16.695 | 56.050 | 27.256 |
| 14.494 | 50.295 | 35.212 |
| 17.580 | 47.299 | 35.121 |
| 20.327 | 43.817 | 35.856 |
| 19.494 | 49.237 | 31.269 |
| 22.566 | 50.463 | 26.970 |

The study area was put into development in 2012, collated the annual production data, and drew the water saturation map of different periods, as shown in the Fig. 8.

| 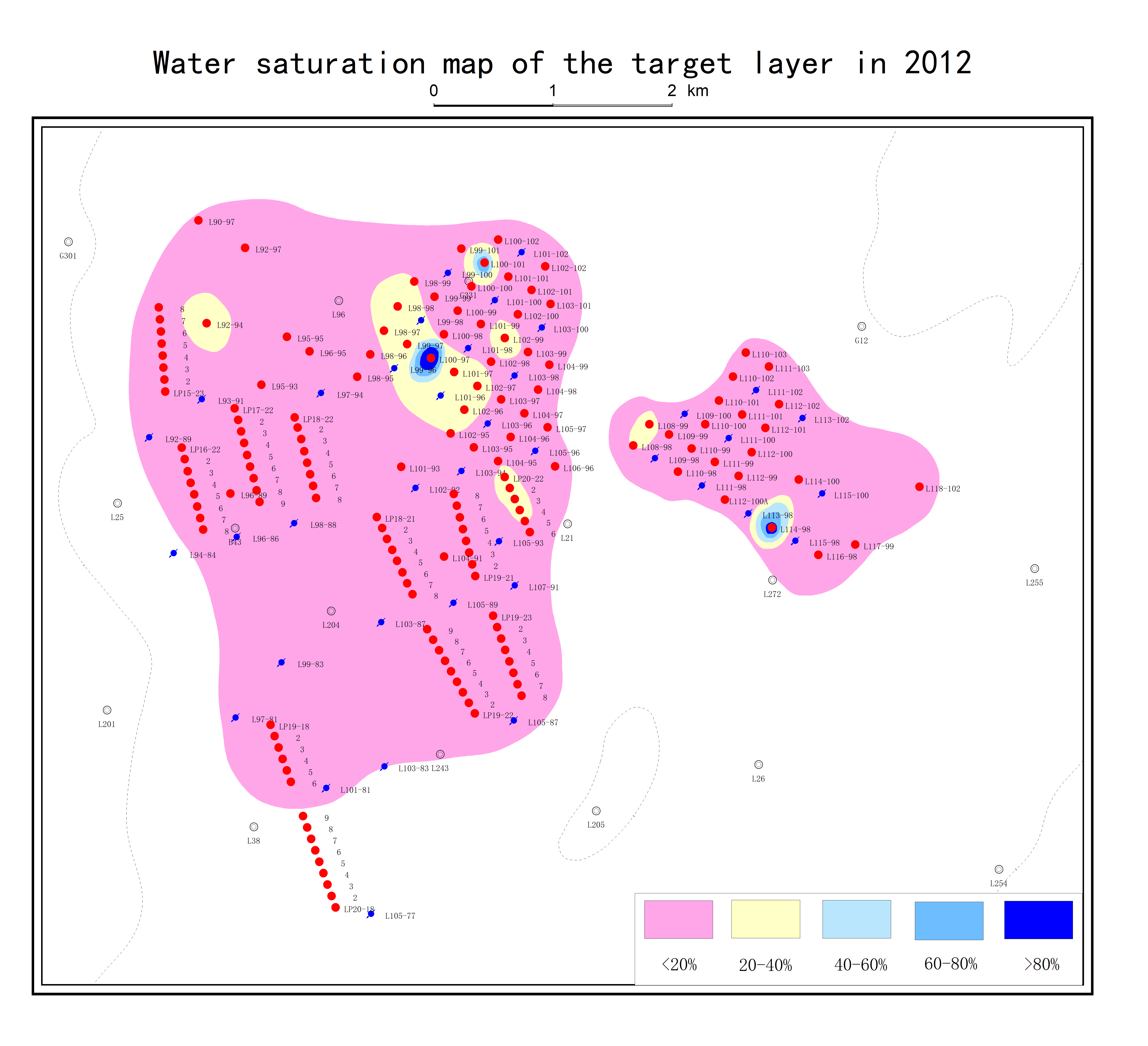 | 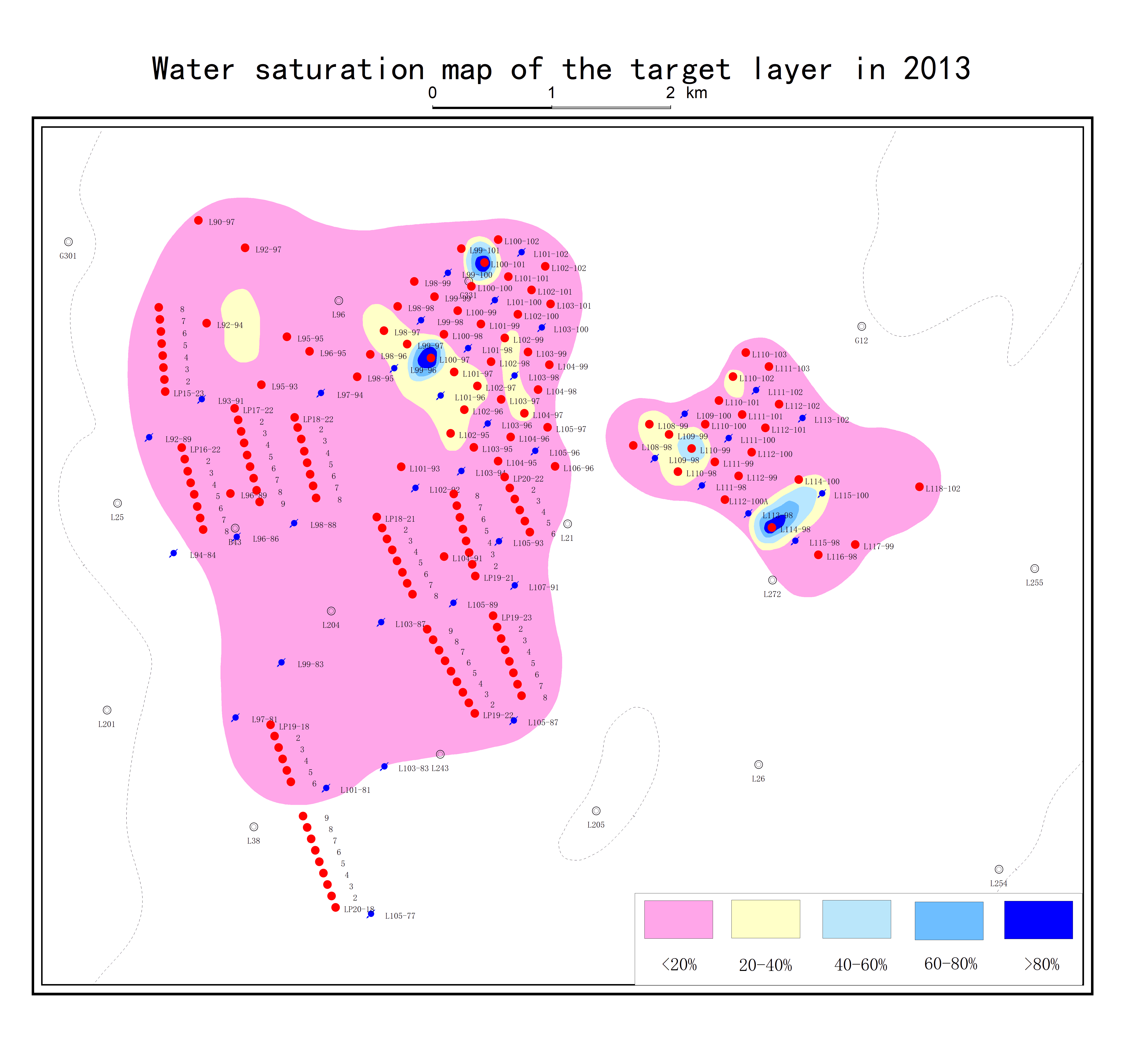 |
| --- | --- |
| Water-cut in 2012 | Water-cut in 2013 |
| 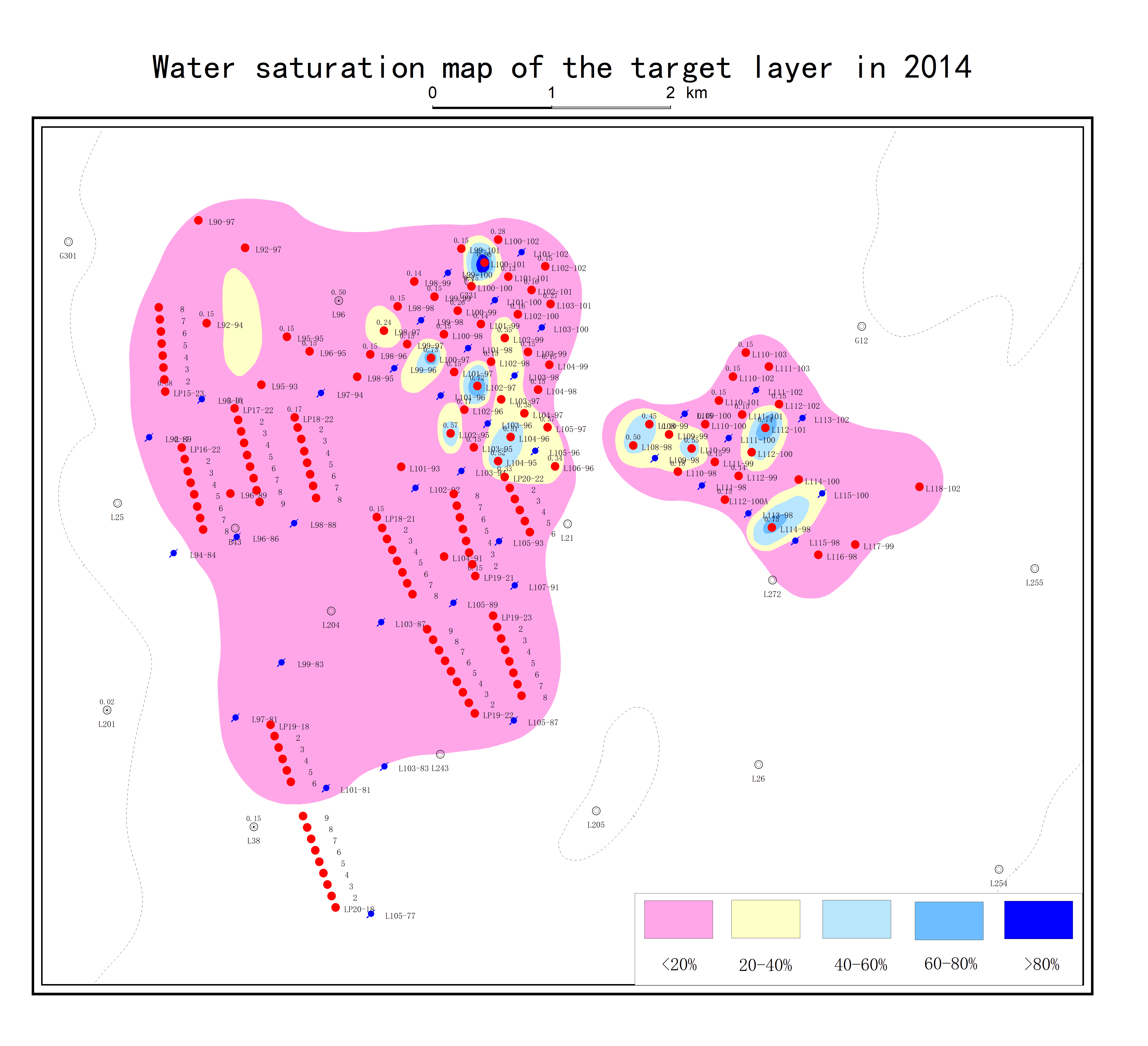 | 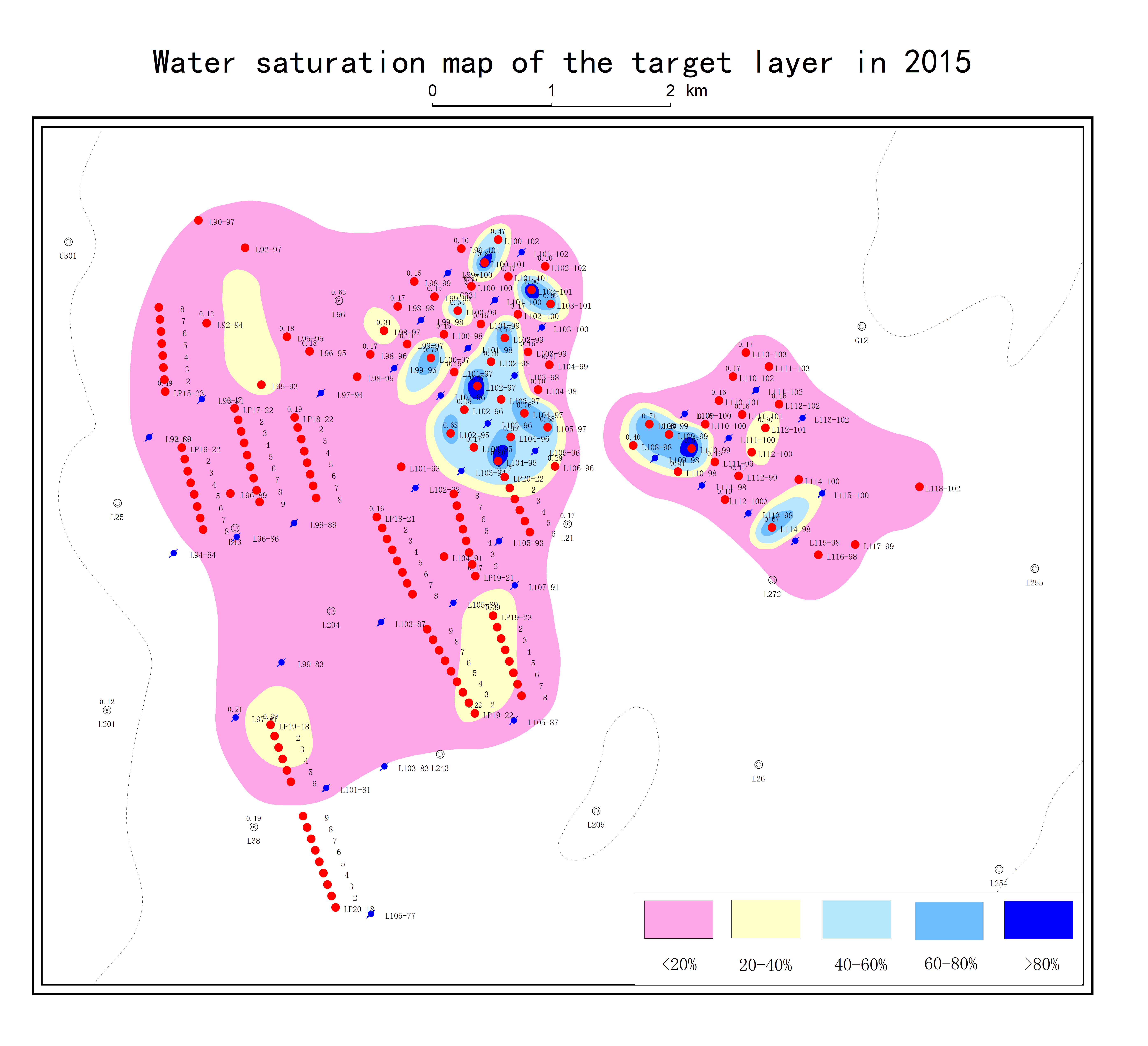 |
| Water-cut in 2014 | Water-cut in 2015 |
| 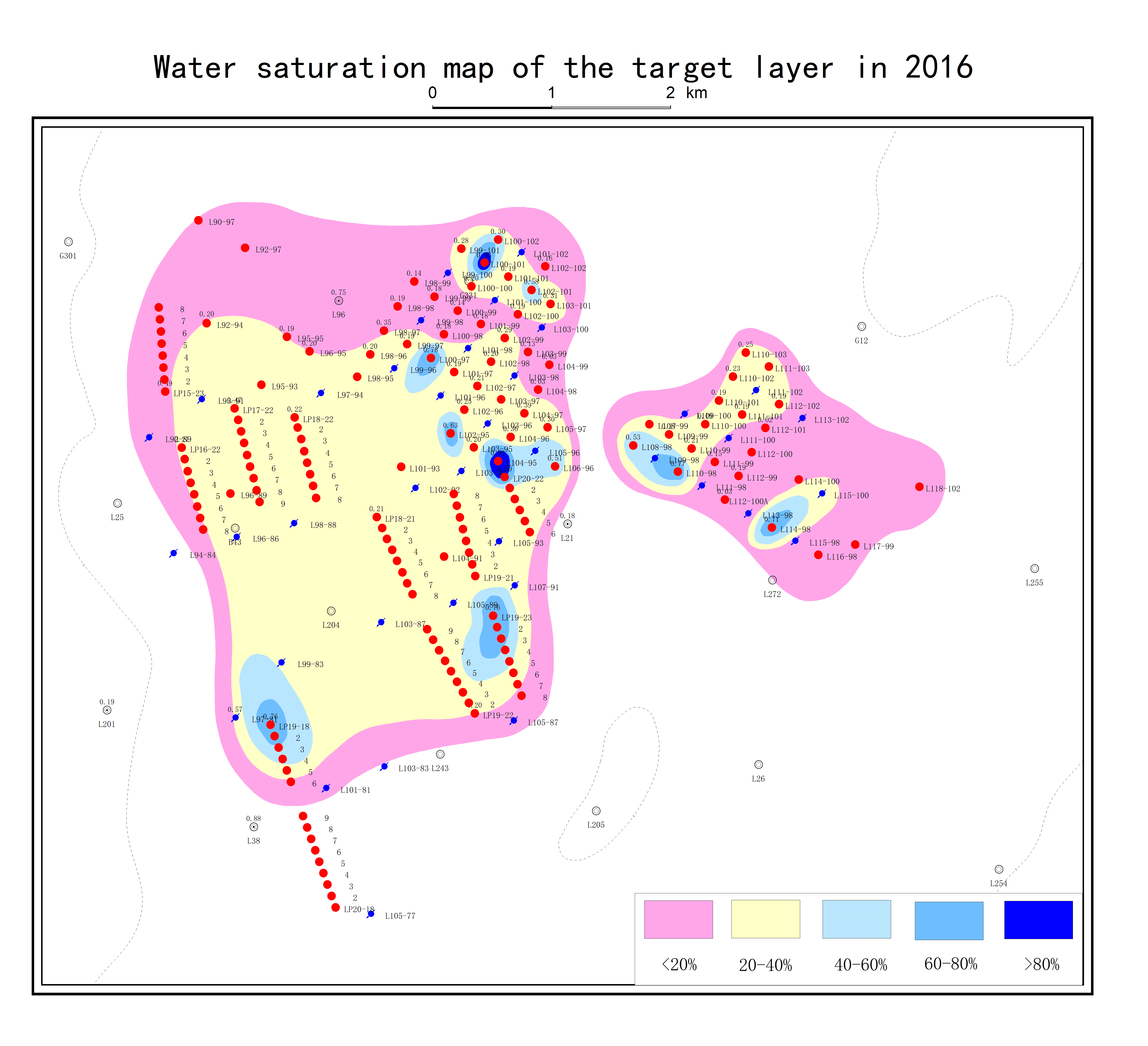 | 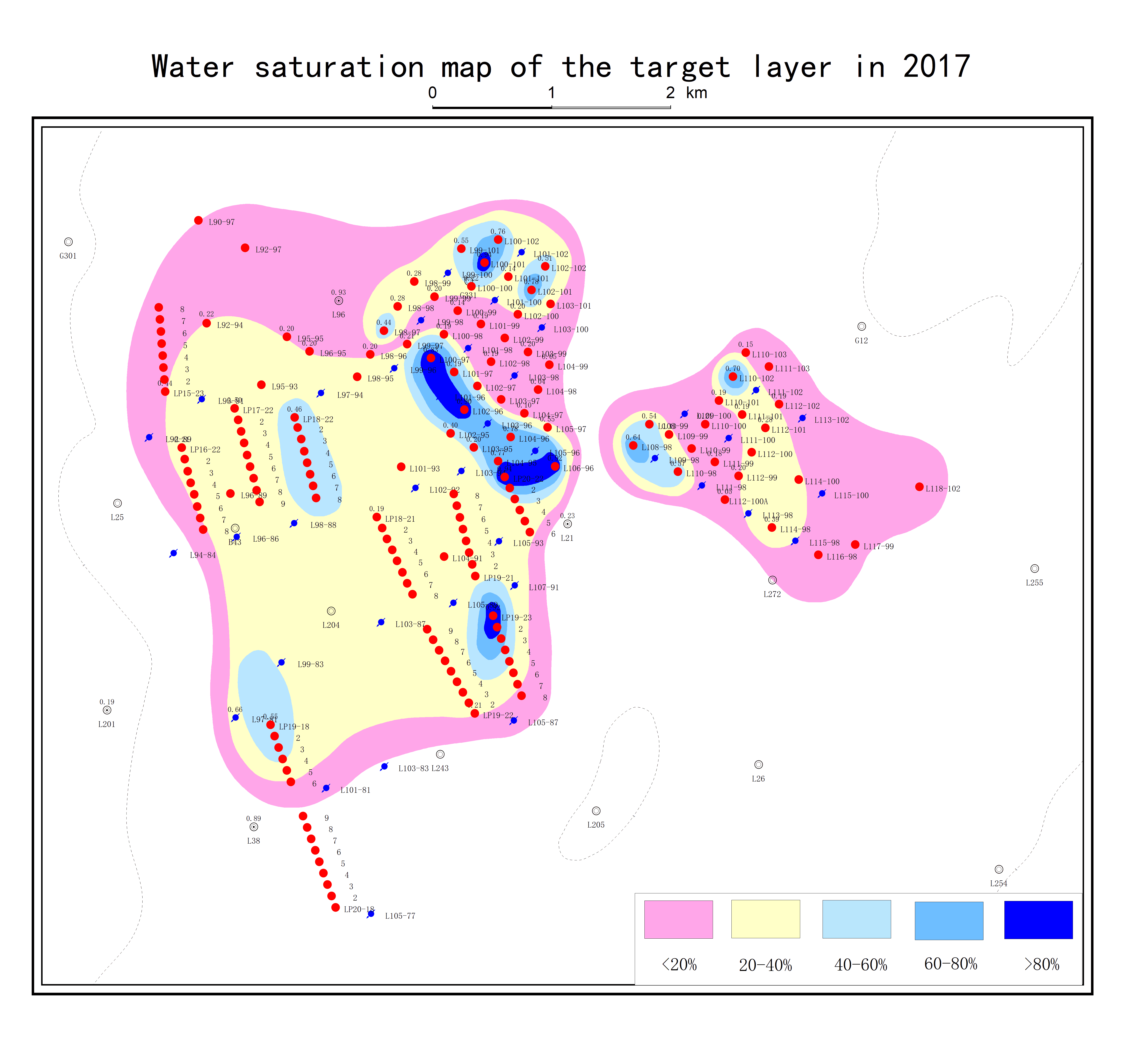 |
| Water-cut in 2016 | Water-cut in 2017 |
| 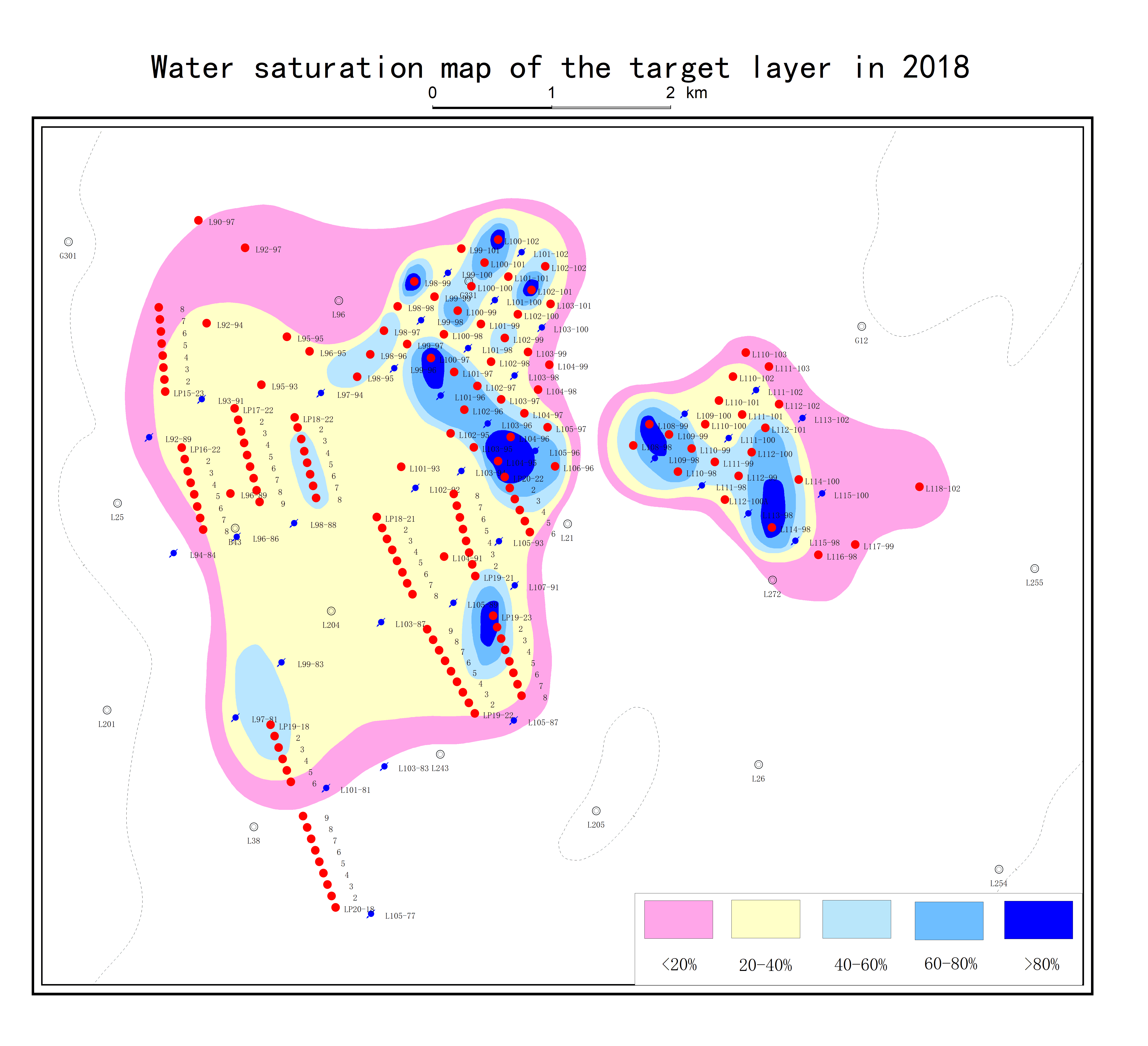 | |
| Water-cut in 2018 | |

Fig. 8 The water saturation contour map of study area. Created using GeoMap v3.6(http://www.jurassic.com.cn/)

1. Corresponding author: Yushuang Zhu, Professor, Doctoral Supervisor, Northwest University, China.

   E-mail address: petroleum_gas@163.com. [↑](#footnote-ref-1)
